# Expressions for Bayesian confidence of drift diffusion observers in fluctuating stimuli tasks

**DOI:** 10.1016/j.jmp.2023.102815

**Published:** 2023-12

**Authors:** Joshua Calder-Travis, Rafal Bogacz, Nick Yeung

**Affiliations:** a Department of Experimental Psychology, University of Oxford, UK; b MRC Brain Network Dynamics Unit, Nuffield Department of Clinical Neuroscience, University of Oxford, UK

**Keywords:** Perceptual decisions, Confidence, DDM, Bayesian, Fluctuating stimuli

## Abstract

We introduce a new approach to modelling decision confidence, with the aim of enabling computationally cheap predictions while taking into account, and thereby exploiting, trial-by-trial variability in stochastically fluctuating stimuli. Using the framework of the drift diffusion model of decision making, along with time-dependent thresholds and the idea of a Bayesian confidence readout, we derive expressions for the probability distribution over confidence reports. In line with current models of confidence, the derivations allow for the accumulation of “pipeline” evidence that has been received but not processed by the time of response, the effect of drift rate variability, and metacognitive noise. The expressions are valid for stimuli that change over the course of a trial with normally-distributed fluctuations in the evidence they provide. A number of approximations are made to arrive at the final expressions, and we test all approximations via simulation. The derived expressions contain only a small number of standard functions, and require evaluating only once per trial, making trial-by-trial modelling of confidence data in stochastically fluctuating stimuli tasks more feasible. We conclude by using the expressions to gain insight into the confidence of optimal observers, and empirically observed patterns.

## 1 Introduction

How humans and other animals make perceptual decisions is of fundamental interest. It is increasingly recognised that decision confidence, an estimate of the probability a decision was correct, is both theoretically important and used in a variety of ways to shape individual and group decision making (Bahrami et al., 2010; van den Berg, Zylberberg, et al., 2016; Desender et al., 2019, 2018; Drugowitsch et al., 2019; Sanders et al., 2016). Confidence has also been linked to psychological disorder (Hauser et al., 2017; Rouault et al., 2018). Reflecting the significance of confidence judgements, substantial efforts have been made to characterise their underlying computational mechanisms (e.g. Balsdon et al., 2020; van den Berg, Anandalingam, et al., 2016; Desender et al., 2020; Fleming & Daw, 2017; Geurts et al., 2022; Kiani et al., 2014; Moran et al., 2015; Pleskac & Busemeyer, 2010; Ratcliff & Starns, 2009; Yu et al., 2015; Zylberberg et al., 2012). The present work builds on these efforts to introduce a set of mathematical expressions for confidence using the drift diffusion model (also known as the diffusion decision model; DDM; Ratcliff & McKoon, 2008), coupled with a Bayesian readout for confidence (Kiani & Shadlen, 2009; Moreno-Bote, 2010; Sanders et al., 2016). The novelty of our contribution is our combination of three aims: To derive expressions for confidence within normatively prescribed frameworks for decision making and metacognitive evaluation; to go beyond simply fitting to aggregated confidence reports and instead develop methods capable of tractable fits to (and predictions about) individual confidence reports (Park et al., 2016); and to provide a flexible modelling framework that can incorporate (and estimate the impact of) several factors recently shown or hypothesised to influence confidence reports. A key feature of our approach is that we derive expressions for the probability distribution over confidence reports (given decisions and response times) rather than focusing on first-order decisions and response-times themselves. Such expressions can be used as the basis for model fitting and parameter estimation, while avoiding the computational cost of making trial-by-trial predictions for decisions and response times (Ratcliff, 1980; Shan et al., 2019; Smith, 2000; Smith & Ratcliff, 2022).

It is important to ask at the outset why we would want to derive explicit mathematical expressions that are computationally cheap to evaluate, when computational modelling can often be performed in other ways. This exercise has two main purposes: We aim for computationally cheap predictions to make trial-by-trial modelling of stochastically fluctuating stimuli feasible, and we aim for explicit mathematical expressions to gain deeper insight into the nature of confidence in an important model. Explicit mathematical expressions provide immediate knowledge of the relationships between different parameters and variables, and how they combine to produce confidence. Indeed, we will see that we can go further and interpret such expressions to understand why different variables have the effect they do (Section 4). Regarding our other motivation, making trial-by-trial modelling feasible, such modelling allows us to capitalise on variability in stimuli, rather than ignoring it or treating it as noise (Park et al., 2016). When modelling on a trial-by-trial basis, a model that can capture the specific effects of each stimulus will outperform a model that can only capture general patterns in confidence across conditions (such as condition means or distributions of aggregated confidence reports). Hence, computationally cheap expressions may facilitate the development and testing of models for confidence that make increasingly precise predictions for behaviour.

The DDM is one of the most prominent models of two-alternative decisions, from a family of models in which observers receive noisy measurements of evidence for the two options (Bogacz et al., 2006; Green & Swets, 1966; Ratcliff & McKoon, 2008). In the DDM, observers track the difference in evidence measurements between the two options. That is, for each sample, observers subtract the measurement for option B from the measurement for option A, and add this to a running total (Ratcliff & McKoon, 2008). When the accumulator tracking this difference reaches a fixed threshold (positive or negative), a response is triggered. The DDM has successfully been used to model decisions in a wide range of tasks (Ratcliff et al., 2016).

The DDM is also a normative model of decision making. By “normative” and “optimal” we refer to observers that, using the information assumed available to them, maximise reward rate (Rahnev & Denison, 2018). Under certain conditions, such as evidence measurements for the two options being equally reliable and signal strength known, the DDM is equivalent to tracking the posterior probability of each option until a fixed threshold on these probabilities is reached (Bitzer et al., 2014; Gold & Shadlen, 2007; Moran, 2015). In such a context, this policy maximises reward rate (Moran, 2015; Wald & Wolfowitz, 1948). When signal strength is unknown, a time-dependent threshold is required, but under standard assumptions it nevertheless remains optimal to track the difference between the two accumulators (Drugowitsch et al., 2012; Moran, 2015; Tajima et al., 2019). For an intuition of why this policy is optimal, consider a case in which the observer has not made a decision after lengthy deliberation. The observer must be accumulating evidence very slowly, suggesting to them that signal strength is very low. If the observer thinks signal strength is very low, there is almost nothing to gain from collecting more evidence measurements, so they should lower their decision threshold and make an immediate decision (Malhotra et al., 2017).

Although the DDM has optimal characteristics, and has been successfully applied to a wide range of decisions (Ratcliff et al., 2016), it is not clear the DDM provides an adequate account of confidence reports. Different ways of modelling confidence using the DDM have been proposed (Yeung & Summerfield, 2014). In one set of models, observers use some form of heuristic, based on variables which are directly accessible in the DDM, such as the state of the accumulator (Pleskac & Busemeyer, 2010), or the time taken to make a decision (Zylberberg et al., 2012). Another approach is to assume that observers map the state of the accumulator, and the time spent accumulating evidence, to the probability they are correct (Kiani et al., 2014; Kiani & Shadlen, 2009; Moreno-Bote, 2010). A Bayesian readout of this kind could be learned over time, through the association of accumulator state and time with success or failure (Kiani & Shadlen, 2009). Alternatively, a Bayesian readout could reflect a probabilistic inference made using knowledge of the statistical structure of the task. One detail to consider is that the confidence readout could be based on a separate evidence accumulator to the one used for the decision, or on multiple evidence accumulators (Balsdon et al., 2020; Fleming & Daw, 2017; Ganupuru et al., 2019; Jang et al., 2012; Ratcliff & Starns, 2009, 2013). However, here we make the simplest assumption that decisions and confidence are based on the same, single, normative, evidence accumulator (Moreno-Bote, 2010).

There are several techniques that can be used to calculate the probability, according to the DDM, of different responses and response times (Brown et al., 2006; Chang & Cooper, 1970; Cox & Miller, 1965; Diederich & Busemeyer, 2003; Drugowitsch, 2016; Navarro & Fuss, 2009; Shinn et al., 2020; Smith, 2000; Tuerlinckx, 2004; Tuerlinckx et al., 2001; Voss & Voss, 2008). Importantly, approaches have been developed that can handle dynamic stimuli (stimuli that change over the course of a trial) and time-dependent thresholds. One approach involves using finite difference methods to approximate the evolution of the probability distribution over accumulator state (which reflects the accumulated difference in evidence measurements; Chang & Cooper, 1970; Shinn et al., 2020; Voss & Voss, 2008; Zylberberg et al., 2018). Time and space are discretised and, working forward from the first time step, we solve a set of simultaneous equations at each time step to find the evolution of the probability distribution over accumulator state. If we are only interested in the probability distribution over response times and choices, we can use expressions described by Smith (2000). To evaluate these expressions we only need to discretise time, not space. Again working forward from the first time step, we can calculate the probability of deciding at each time step. Both approaches require that we discretise the time course of a trial into small time steps, and the number of computations required will scale with the number of time steps considered. Hence, in both approaches, we must perform a large number of computations (unless we use a task and stimulus in which modelling with large time steps is justified; Park et al. (2016)).

This computational cost becomes important if we want to leverage the trial-by-trial variability inherent in stimuli that fluctuate stochastically within individual trials. (Practical solutions for trial-by-trial modelling already exist for static stimuli under certain conditions; e.g. Wiecki et al. (2013).) Often the computation time needed for calculating predictions is reduced by making predictions on a condition-by-condition basis (Park et al., 2016; e.g. van den Berg, Anandalingam, et al., 2016; Kiani et al., 2014; Ratcliff & McKoon, 2008; Zylberberg et al., 2016). We design the experiment so that there are a small number of different conditions (e.g., levels of stimulus contrast), then we treat all trials from a single condition as the same, and make predictions for behaviour in each condition, rather than for each stimulus individually. This approach does not capitalise on the trial-by-trial variability of the stochastically fluctuating stimuli that are often used (for model-free analyses that do capitalise on trial-by-trial variability see Charles and Yeung (2019), Kiani et al. (2008), Zylberberg et al. (2012)). A method that reduced the computational cost of making trial-by-trial predictions for stochastically fluctuating stimuli could allow us to perform model fitting that capitalises on rather than discards the rich data produced from such stimuli.

Another approach is to derive explicit mathematical expressions for model predictions, which could dramatically reduce computation time. Moreno-Bote (2010) derived expressions for confidence that take into account time-dependent thresholds, and that could be extended to account for stochastically fluctuating stimuli of the kind we consider below. However, these derivations use two assumptions about the computation of confidence which are not in line with recent findings. First, Moreno-Bote (2010) made the intuitive assumption that decisions and confidence are based on the same information. However, as sensory and motor processing takes time, there will be stimulus information in these processing “pipelines” that does not contribute to the initial decision, but that nevertheless informs subsequent confidence judgements (van den Berg, Anandalingam, et al., 2016; Charles & Yeung, 2019; Moran et al., 2015; Ratcliff & McKoon, 2008; Resulaj et al., 2009; Yu et al., 2015). For example, Yu et al. (2015) found evidence that stimulus processing following a decision contributes to confidence, regardless of whether or not the stimulus continues to be presented, consistent with a processing pipeline. Moreover, information in the processing pipeline is affected by trial-to-trial fluctuations in signal strength, and this may have important effects on confidence (Pleskac & Busemeyer, 2010). Second, there is now substantial evidence that the process that “reads out” confidence into a behavioural report is corrupted by “metacognitive noise” (Bang et al., 2019; van den Berg et al., 2017; De Martino et al., 2013; Maniscalco & Lau, 2012, 2016). This additional noise in the readout may reflect imperfections in the transfer of information between decision and metacognitive processes (Bang et al., 2019; De Martino et al., 2013), or may be because confidence reports themselves are sensitive to factors outside of the first-order decision process (Maniscalco et al., 2017; Rahnev et al., 2015; Shekhar & Rahnev, 2020). We aim for mathematical expressions that take these important features into account.

**Fig. 1:**
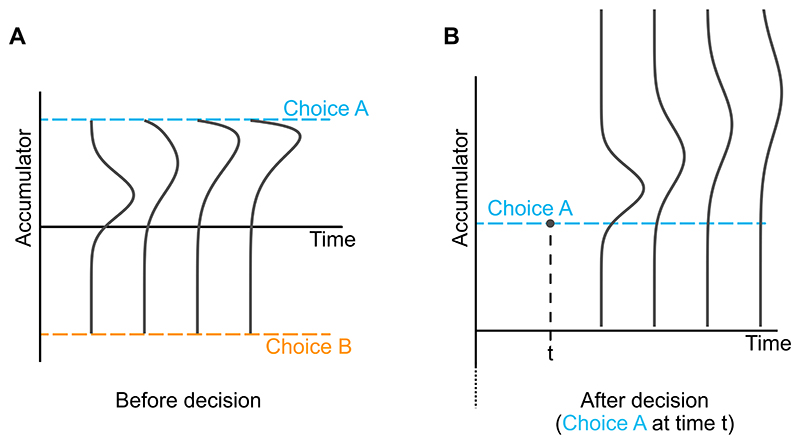
Probability distributions over accumulator state before and after a decision. Even if increments in the accumulator are normally distributed, prior to a decision the probability distribution over the state of the accumulator quickly becomes non-normal. This is because, if we get to time *t* without a decision, we know the accumulator has not been beyond either decision threshold prior to *t*. Following a decision (the time of which is represented by the dot in the right panel), normally distributed increments in the accumulator lead to a normal distribution over accumulator state, because there are no longer decision thresholds.

A key idea that affects the scope of our derivations is that, whereas it may be very difficult or impossible to derive simple expressions for decisions and response times, it may be much simpler to find expressions for confidence (given a specific decision and response time). Prior to a decision, even if changes to the state of the accumulator are normally distributed over small intervals of time, the probability distribution over the state of the accumulator will not be normal. This is because, having reached a time *t* without a response, we know that the accumulator is not beyond either threshold, nor has it been at any point up to *t* (otherwise the observer would already have made a decision; Moreno-Bote (2010)). This constraint results in non-normal probability distributions over accumulator state (Fig. 1A), with associated mathematical expressions that either feature infinite summations, or may even be intractable (Ratcliff, 1980). In contrast, it is much simpler to express confidence directly, in terms of the evolving state of the accumulator following a decision. Either on the basis of the central limit theorem, or the results of Ratcliff (1980), we expect a normal distribution over the state of the evidence accumulator when decision thresholds are absent, even if evidence signal strength varies within a trial (as would be the case for stochastically fluctuating stimuli). We build on this work by considering confidence in the related situation of evidence accumulation following a decision threshold crossing. This situation turns out to be more complex, nevertheless – following a decision – decision boundaries are no longer relevant, hence normally distributed changes in the state of the accumulator lead to a normal distribution over this state (Fig. 1B). Crucially, our aim will be to find probability distributions for confidence, given that a specific decision was made at a specific time, i.e., given that a decision threshold has already been crossed. As a result, we will not have to take into account the non-normal probability distributions that characterise non-terminated decision processes. Thus, our contribution is not to provide new expressions for the probability distribution over response times and decisions in diffusion models. We aim to bypass the complexities associated with response times and decisions, and instead focus on confidence.

Using this strategy we derive approximate expressions for the probability, according to the DDM, of different confidence reports, given the response and response time on a trial. As discussed, the framework of the DDM with possibly time-dependent thresholds, includes (under standard assumptions) the optimal decision policy, whether or not signal strength is known by the observer. Confidence is allowed to be a noisy readout of the probability of being correct, and we account for the effects of pipeline evidence, and variability in signal-to-noise ratio. The derived expressions only need evaluating once per trial, instead of at each very small time step within each trial, and allow for stochastically fluctuating stimuli of a certain form, thereby making trial-by-trial modelling of such stimuli feasible. As discussed, making trial-by-trial modelling feasible is one of our main aims. Once we have derived mathematical expressions for confidence, we will additionally be able to use them to gain deeper insight into the nature of confidence within the framework of the normative DDM.

## 2 Model

### Overview

Our aim is to derive expressions for the probability distribution over confidence, given the response and response time on a trial. To derive these predictions we must first specify a model for decisions and confidence, and the context in which that model is to be applied. The equations in the following subsections formalise a generic two-alternative decision making task with stochastically fluctuating evidence, and specify a model that conforms to the well-established ideas of the DDM (Ratcliff & McKoon, 2008; Ratcliff et al., 2016). This DDM-based model features some natural extensions – inspired by previous work – to deal with the possibility of stochastically fluctuating evidence (Drugowitsch et al., 2012; Ratcliff, 1980; Smith, 2000). Finally, a confidence readout is specified, which is based on the common idea that observers read out the probability they are correct (Kiani et al., 2014; Moran, 2015; Pew, 1969; Sanders et al., 2016).

**Fig. 2:**
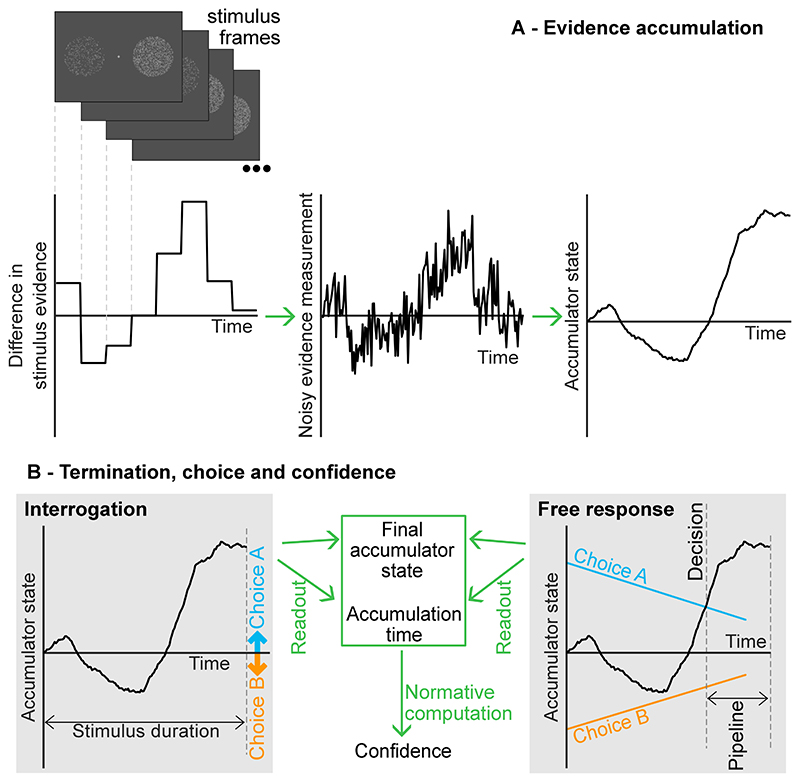
The model of confidence and decisions. Stochastically fluctuating evidence is presented for the two response options. For example, the observer may be presented with two clouds of dots that fluctuate each stimulus frame, and have the task of determining which cloud contains more dots on average. The observer receives noisy measurements of the difference in stimulus evidence, and accumulates these measurements to make their choice. In the interrogation condition, the duration of the stimulus is set by the researcher. The observer accumulates noisy measurements until the stimulus ends and all evidence is processed. Then the observer simply picks the option that is favoured by the accumulated measurements. In the free response condition, the observer uses decision thresholds, one for each option, which trigger a response. Following a decision, evidence measurements in the processing pipeline are accumulated, and confidence is informed by the accumulator state at the time of threshold crossing, plus changes to the accumulator caused by evidence measurements from the pipeline.

We consider a situation in which observers must make a choice between two alternatives. The presented stimulus provides two evidence signals (i.e., the stimulus contains two decision-relevant features), one for each option, and the evidence provided by the stimulus (i.e., the decision-relevant features) can fluctuate stochastically over time within a trial (Fig. 2; Bogacz et al., 2006; Moreno-Bote, 2010). For example, the observer might be presented with two clouds of dots, with the number of dots in each cloud constantly changing. Their task might be to determine which of the two clouds contains the most dots on average (Charles & Yeung, 2019; Pleskac et al., 2023, 2019; Zeigenfuse et al., 2014). Here the dots in the two clouds would correspond to the two streams of evidence. We assume that the observer only receives noisy measurements of the presented evidence (Green & Swets, 1966; Ratcliff & McKoon, 2008). In our model, consistent with the DDM, the observer takes the difference between the noisy measurements of evidence for the two options, and accumulates this difference (Fig. 2).

A further point worth mentioning at the outset is that we only aim to model a certain kind of stimulus. We focus on stimuli, and stimuli durations, for which perceptual integration can plausibly be ignored. As a result, we do not model a perceptual integration stage that operates on the perceptual input (e.g., by applying a low pass filter) before it enters the evidence accumulation (Smith & Ratcliff, 2009). The question of whether perceptual integration needs to be modelled has been explored in the context of response and response time models, and the answer appears to depend on the task used (Smith & Ratcliff, 2022). For example, Smith and Ratcliff (2022) found no strong evidence for extended perceptual integration in tasks involving 20 Hz flashing grids of squares, where the aim was to determine the predominant colour or brightness, whereas Smith and Lilburn (2020) reported that accounting for the effects of perceptual integration leads to better fitting models for the random dot motion task (see Kiani et al., 2008). We return to this point in the discussion.

Typically, following stimulus onset, a participant can respond whenever they wish. Some instruction or incentive may be given to respond in a certain way, such as fast and accurately, but beyond this the participant is free to set the time of response (e.g. Ratcliff & McKoon, 2008). The stimulus continues to be presented until a response is made. We call this condition “free response” (but it is also referred to as the “information controlled” condition elsewhere; Bogacz et al. (2006), McMillen and Holmes (2006), Ratcliff (1980)). Following the DDM, we assume the observer sets two thresholds on the accumulator state, one for each choice (Bogacz et al., 2006; Ratcliff & McKoon, 2008). When the accumulator reaches one of these thresholds, the corresponding response is triggered (Fig. 2). As discussed in Section 1, measurements corresponding to evidence that has recently been presented will still be in sensory and motor processing pipelines at the time of response, and hence will not contribute to a decision (Resulaj et al., 2009). These measurements will be processed immediately following a response, and will be used to inform confidence (van den Berg, Anandalingam, et al., 2016).

We also consider the “interrogation” condition (McMillen & Holmes, 2006), where the observer must respond at a time controlled by the researcher. (This and closely related conditions are also referred to as “time controlled” and “response signal” conditions; Dosher (1976), Ratcliff (1980, 2006), Schouten and Bekker (1967), Usher and McClelland (2001).) In this case the stimulus is presented for a finite amount of time. Before the stimulus clears the participant cannot respond. Once the stimulus clears, the observer uses the final state of the accumulator (which reflects all evidence presented in the stimulus) to determine their response (Fig. 2; Bogacz et al., 2006). Although we will focus on this decision making strategy, which is normative in the absence of a cost associated with accumulating evidence (Bogacz et al., 2006), we note that an alternative modelling choice would be to also include the idea of decision thresholds in the interrogation condition, assuming that if a decision threshold is reached people stop accumulating evidence and withhold their response until the appropriate time (Balsdon et al., 2020; Ratcliff, 2006). Such a strategy would become normative if there was an intrinsic cost to accumulating evidence (beyond the cost associated with spending time on the task, which is not under the control of the observer in this condition; Kiani et al., 2008). There is evidence that careful selection of when response times are enforced in the interrogation condition can minimise this possibility (Rosenbaum et al., 2022). In both the free response and interrogation conditions, the observer uses a Bayesian readout of confidence which depends on the final state of the accumulator once all evidence has been processed, and the time spent accumulating evidence (Kiani et al., 2014; Moreno-Bote, 2010).

In the following subsections we first describe the observer’s task mathematically, before looking at the rule a Bayesian observer would use to map evidence measurements to a decision and confidence. Finally, we describe the noisy “read out” process which determines confidence reports (Fleming & Daw, 2017). In Section 3 we turn to the main aim of the paper, which is to use the drift diffusion framework to derive simple expressions for the probability distribution over confidence reports, given a response and response time. A summary of symbols used in the derivations can be found in Table 1. We use the convention that log refers to the natural logarithm.

### Task

The observer’s task is to determine the correct response, by inferring which evidence stream (i.e., which decision-relevant feature of the stimulus) is drawn from the distribution with the greater mean (Bogacz et al., 2006; Moreno-Bote, 2010). As we are considering a DDM observer who tracks the difference in the evidence measurements for the two alternatives, we only need to consider the difference in evidence provided by the two evidence signals from the stimulus.

We consider the case where the two options are equally probable. Denote the mean evidence for the two options, *μ_1_* and *μ_2_*, and the difference between these means, *μ = μ_2_ - μ_1_*. These values are fixed throughout an individual trial. We consider a situation in which the absolute value of the difference between the two means is the same for all trials. However, we incorporate variability in signal strength below (see discussion of variability in drift-rate scaling, *φ*). Let Δ*μ* denote a fixed positive value which determines the absolute value of the difference between the two means. This setup gives us,

(1) $$p(S=1)=p(S=2)=\frac{1}{2}$$

(2) $$\mu =\{\begin{array}{ll} -\text{Δ}\mu & \text{if}\;S=1\\ \text{Δ}\mu & \text{if}\;S=2 \end{array}.$$

*S* denotes the stimulus (1 or 2) with the greater mean evidence, and hence the correct answer.

Denote the total time during which the stimulus is presented *t_e_*, and the time of the response relative to the beginning of the trial *t_r_*. In the free response condition *t_e_ = t_r_* because a response triggers the end of the stimulus. For the free response condition we have to consider the effects of the decision thresholds (discussed above). Denote the time spent accumulating measurements prior to the first crossing of a decision threshold, *t_d_*. A threshold crossing triggers a response. However, because of delays in sensory and motor processing, there is a lag between the point of internal commitment to a decision and the point at which that decision is externally registered via an overt movement, and even at the point of the overt response some recently received sensory evidence will still be being processed (Luce, 1986; Resulaj et al., 2009). Hence, information presented in the stimulus immediately prior to the response will not contribute to the decision. Denote the duration of the stimulus immediately prior to the response that does not contribute to the decision, because corresponding measurements are still in processing pipelines, *I*. The time spent accumulating evidence until the first decision threshold crossing and the duration of the processing pipeline, will together equal the time taken to respond, *t_r_ = t_d_* + *I*.

In the interrogation condition the observer uses all information presented in the stimulus – by accumulating evidence for a duration equal to the duration of evidence presentation – to determine both their response and confidence (Bogacz et al., 2006). Once all evidence has been accumulated, after *t_e_*, a response is then triggered. We make the natural assumption that sensory and motor processing delays are of the same duration in both the interrogation and free response conditions, although in the interrogation condition no further information is gathered during this time because the stimulus is no longer being presented. In this condition, response time is therefore given by *t_r_ = t_e_* + *I*.

We consider here the general case of a stimulus that provides evidence that varies stochastically over time within a trial (Fig. 2). Our derivations also apply to constant evidence as a special case of fluctuating evidence. We consider evidence (conveyed through decisionrelevant features of the stimulus) that is piecewise-constant within short stimulus “frames” of duration *t_f_*. During each short stimulus frame the stimulus is static, and hence so too are the decision-relevant features of the stimulus, and the evidence provided by the stimulus. We use *E_i_* to denote the evidence presented for option 2 minus the evidence presented for option 1 (i.e., the difference in evidence) during frame *i*. If the difference in evidence presented in each frame is normally distributed around the underlying mean, *μ*, then we have,

(3) $$p(E_{i}|\mu )=N(E_{i};\mu ,\sigma_{E}^{2}),$$

where $\sigma_{E}^{2}$ is the variance over the presented difference-in-evidence. Note that *μ* does not correspond to anything the observer directly observes. As discussed, *μ* is the underlying mean difference-in-evidence presented for the two options, and is constant throughout a trial. The actual presented evidence, *E_i_*, is what varies over the course of a trial (or more precisely it varies from stimulus frame-to-frame), and is drawn from a distribution centred on the underlying means.

As in the DDM, the presented evidence drives an internal evidence accumulation that is subject to normally distributed noise (Ratcliff & McKoon, 2008). We also take into account the fact that the stimulus fluctuates stochastically within each trial: Each stimulus frame drives the evidence accumulation for a duration equal to the duration that frame is presented for. As discussed above, we assume the effects of any pre-decision perceptual integration stage can be ignored (Smith & Ratcliff, 2022). Consider a discretisation of time into very short time steps (much shorter than the duration of a frame) of duration *δt*. Over a small time step, *j*, the incremental change in the state of the accumulator tracking the difference in evidence measurements, denoted *δx_j_*, can be described by,

(4) $$p(\delta x_{j}|\delta E_{j},\varphi )=N(\delta x_{j};\delta E_{j}\varphi ,\sigma_{acc}^{2}\delta t),$$

where $\sigma_{acc}^{2}$ is the variance of noise in the accumulation (Drugowitsch et al., 2012). *δE_j_* indicates the difference-in-evidence presented in time step *j* only, not over the course of a frame, and is determined by the stimulus frame currently being processed (*E_i_ = ∑_j_ δE_j_* where the summation is taken over all the time steps in a stimulus frame). *φ* is a random variable which accounts for variability in drift rate. “Drift rate” refers to the rate at which evidence presented in the stimulus drives the accumulation of evidence measurements (Ratcliff & McKoon, 2008). Where a stimulus, and hence drift rate, is constant over the course of a trial, drift rate variability is trial-to-trial variability in this rate (Ratcliff, 1978; Ratcliff & McKoon, 2008; Ratcliff & Smith, 2004; Voskuilen et al., 2016). Here, where stimulus evidence, and hence drift rate, fluctuate stochastically over the course of each trial, we operationalise drift rate variability as a multiplicative factor that determines how well stimulus information is processed. To distinguish this operationalisation from the usual operationalisation, we sometimes refer to the multiplicative factor *φ* as the “drift-rate scaling”, because we set the mean value of this variable to be one regardless of the strength of the presented evidence. When the drift-rate scaling is high, the signal extracted from the stimulus is greater, and evidence is accumulated rapidly. Noise in the accumulation is unaffected, hence, a higher drift-rate scaling also leads to a higher signal-to-noise ratio.

It is usually assumed that drift rate variability follows a normal distribution (Ratcliff, 1978; Ratcliff & McKoon, 2008). We make the same assumption here,

(5) $$p(\varphi )=N(\varphi ;1,\sigma_{\varphi }^{2}).$$

To be clear, drift-rate scaling is constant throughout an individual trial, but may vary from trial-to-trial.

### Observer

This largely standard task setup generates a standard inference problem for observers, that can be summarised as follows (derivation in Supplement Section B). Observers are aiming to infer *S*, which affects the average rate of evidence accumulation, *ν* (Table 1), via

(6) $$p(v|S=1)=N(\nu ;-v_{0},\sigma_{v}^{2})$$

(7) $$p(\nu |S=2)=N(\nu ;v_{0},\sigma_{v}^{2}).$$

Increments in the evidence accumulation are related to *ν* via,

(8) $$p(\delta x_{i}|\nu )=N(\delta x_{i};v\delta t,s^{2}\delta t).$$

The inference problem described by (6), (7) and (8) does not perfectly match the true generative model. Specifically, to arrive at this inference problem we make the plausible assumptions that (a) observers ignore the fact that evidence (conveyed by decision-relevant features of the stimulus) is constant within each static stimulus frame, and (b) observers ignore the increased effect of variability in the stimulus when the drift-rate scaling is high, and the decreased effect when the drift-rate scaling is low (Supplement Section B). In Supplement Section B we verify that for an observer with such beliefs, confidence and performance remain closely aligned.

Given the above inference problem, the observer can compute a scaled version of the log-posterior ratio, *x_lp_*, using the sum of accumulated evidence $x=\sum_{i=1}^{N}\delta x_{i}$ over all timesteps of the accumulation up to the present timestep, *N*, and the time spent accumulating that evidence, *t* (Supplement Section B),

(9) $$x_{lp}=\frac{x}{\theta (t)},$$

where *θ*() is an abbreviation, and specifically $\theta (t)=(s^{2}+t\sigma_{v}^{2})/(s^{2}+\sigma_{v}^{2}).$

A Bayesian observer would report whichever option is more likely. Hence, they will report *S* = 1 when *x_lp_* < 0, or *x* < 0. Denote this report *R* = 1, and a report for *S* = 2 as *R* = 2. In the interrogation condition, the observer simply has to wait for the stimulus to end at *t_e_*. Once the observer has processed and accumulated all evidence measurements, the observer can respond according to the sign of the final accumulator state, *x* (Fig. 2). In the free response condition, the observer uses a decision threshold for triggering a response (Bogacz et al., 2006; Ratcliff & McKoon, 2008). This threshold describes an absolute value of the accumulator, |*x*|, which triggers a response when reached. We allow the threshold to vary with time (Drugowitsch et al., 2012). As discussed, at the time of response, measurements corresponding to recently presented evidence will still be in the processing pipeline (Resulaj et al., 2009). The response will be based on *x* at the time of the decision, *t_d_*, while confidence will incorporate additional pipeline evidence measurements (Fig. 2). Processing will continue until measurements from the full duration of stimulus presentation, *t_e_*, have been processed.

The (scaled) lot-posterior ratio can be used by observers to compute confidence, because it is monotonically related to the probability that they are correct (Supplement Section B). If the observer reports *R* = 2 then a more positive value of *x_lp_* is associated with a greater probability of being correct. The direction is reversed for *R* =1 choices.

We do not assume that confidence is a direct readout of the (scaled) log-posterior ratio. Instead, we allow the possibility that metacognitive noise corrupts this estimate (De Martino et al., 2013; Maniscalco & Lau, 2012, 2016), and hence that confidence is based on a noisy representation of *x_lp_*, denoted *x_c_*,

(10) $$p(x_{c}|x_{lp})=N(x_{c};x_{lp},\sigma_{m}^{2}),$$

where *σ_m_* is the standard deviation of metacognitive noise. (Note that this noisy readout of the scaled log-posterior ratio, is equivalent to a scaled version of a noisy readout of the unscaled log-posterior ratio; Supplement Section D.)

We would also like to minimise the number of assumptions we make about how *x_c_* is transformed into a confidence report. There is evidence that different people use confidence scales in different ways (Ais et al., 2016; Festinger, 1943; Navajas et al., 2017). To make minimal assumptions about how people view and treat confidence scales, we treat confidence reports, *C*, as ordinal data only (Aitchison et al., 2015). Confidence reports on a continuous scale can be analysed by binning them first.

If people report greater confidence when *x_c_* favours their decision to a greater extent, then all confidence reports falling into a higher confidence bin will have come from further along the *x_c_* scale (in the direction that favours the choice made). Using *d_i_* we denote the boundary on *x_c_* which separates the confidence reports which fall into confidence category *C* = i − 1 from *C* = i, when the observer reports *R* = 2. When *R* =1, the boundary applies to *−x_c_*, or equivalently, a boundary of *−d_i_* is applied to *x_c_*.

## 3 Results

**Fig. 3:**
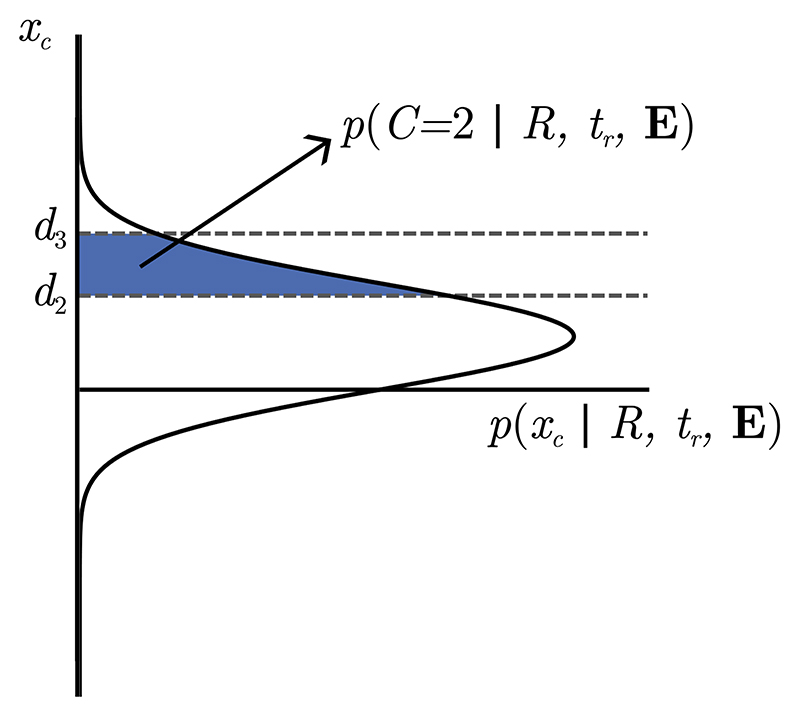
Probability of a confidence report, based on a distribution over *x_c_*. *x_c_* is a noisy representation of the (scaled) log-posterior ratio. We make no specific assumptions about how observers use confidence scales apart from assuming that, if the observer reports higher confidence *C*, then the underlying variable *x_c_* has a greater absolute value, in the direction corresponding to the response made. We use *d_i_* to denote the boundaries between values of *x_c_* that lead to different confidence reports. If we know the distribution over *x_c_*, then the probability of a specific confidence report can be found by integrating *x_c_* or −*x_c_* between the corresponding boundaries. Whether we integrate over *x_c_* or −the limits become*x_c_* depends on the response made.

We now have a complete description of the model, and everything we need to derive the probability distribution over confidence reports in both the interrogation and free response conditions. We would like to find the probability distribution over confidence, given the evidence presented, ***E***, the response, *R*, and in the free response condition, the amount of time the observer monitors the stimulus before making a response, *t_r_*. (***E*** is a vector containing every *E_i_*.) A key variable is the observer’s (scaled) log-posterior ratio after they have seen all evidence, *x_lp_*. Our general strategy will be to find a probability distribution over this variable. From this distribution we will be able to infer a distribution over the noisy readout of the (scaled) log-posterior ratio, *x_c_*. As described in the previous section, on a trial with a response *R* = 2, if *x_c_* falls between *d_i_* and *d_i+1_* the observer reports confidence *C* = *i*. If *R* = 1, the boundaries are *−d_i+1_* and *−d_i_*. The probability of a confidence report *C* = *i* will be given by the probability that *x_c_* falls between the corresponding boundaries (Fig. 3).

Throughout we keep the dependence of the predictions on model parameters implicit: The probability distribution over confidence reports depends not just on the evidence presented, the response, and the time spent monitoring the stimulus, but also the parameters of the model. For the sake of readability this dependence is kept implicit in conditional probabilities in the derivations. (E.g., we write *p*(*C*|*R, t_r_, **E***) instead of *p*(*C*|*R,t_r_, **E**,Ξ*), where *Ξ* represents the set of parameters.) However the parameters on which the predictions depend are of course of great practical importance. It will be these parameters that we adjust as we fit the model to data, and by constraining particular parameters to certain values we will be able to create different variants of the model for comparison. Parameters for fitting to data are listed in Table 2. Decision threshold is listed but this is not in itself a parameter. We will see that the modeller has freedom over what shape decision threshold to use, and how to parameterise this function.

### Interrogation condition

In the following section we present an overview of the derivations for the expressions for confidence in the interrogation condition. The complete derivation is presented in Supplement Section E.

**Fig. 4:**
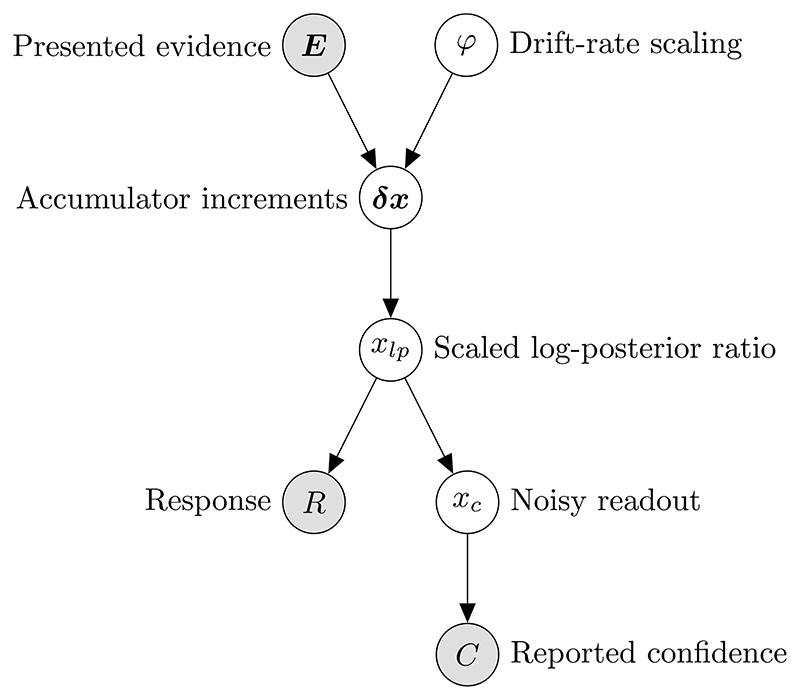
Representation of the generative model for confidence reports in the interrogation condition, from the perspective of the researcher. We want to infer the probability of reported confidence, using our knowledge of the evidence presented and the response given.

In a trial from the interrogation condition, the stimulus is presented for some amount of time, *t_e_*. The observer can only respond after the end of the stimulus. We aim to find the probability of confidence reports, given the response and evidence presented. Assuming that the response occurs at some fixed amount of time following *t_e_*, the response time provides us with no information. This is because *t_e_* is set by the researcher, and hence is unaffected by processes internal to the observer. A summary of the generative model for interrogation condition confidence reports, from the perspective of the researcher, is shown in Fig. 4.

We start by integrating *x_c_* over the region which leads to a confidence report *C = i*. When the response is *R* = 2, this is the region between *d_i_* and *d*_*i*+1_ (Fig. 3). The case where *R* = 1 is identical except the limits become *−d_i+1_* and *−d_i_*. Also marginalising over, *x_lp_* using Bayes rule, and rearranging gives,

(11) $$p(C=i|R=2,E)=\int_{d_{i}}^{d_{i+1}}dx_{c}\frac{\Psi \text{(}x_{c}\text{)}}{p(R=2|E)},$$

where,

(12) $$\Psi (x_{c})=\int dx_{lp}p(x_{c}|x_{lp})p(R|x_{lp})p(x_{lp}|E).$$

An expression for *p*(*x_lp_*|***E***) can be found by marginalising over *φ*, using the independence of ***E*** and *φ* (when not conditioned on other variables; see Fig. 4), and using (4), (5) and (9). Following rearrangement, the resulting expression is,

(13) $$p(x_{lp}|E)=N(x_{lp};\frac{E}{\theta (t_{e})},\frac{E^{2}\sigma_{\varphi }^{2}+t_{e}\sigma_{acc}^{2}}{\theta^{2}(t_{e})}),$$

where *E = ∑_i_ δE_i_* (sum taken over all time steps in all relevant frames), and *t_e_ = ∑_i_ δt* is the duration of evidence presentation.

The simpler case in which there is no metacognitive noise (and hence *x_c_ = x_lp_*) is treated, along with the full derivations, in Supplement Section E. Here we summarise the derivations for the more general case, in which the possibility of metacognitive noise is also taken into account. Nevertheless, it is worth briefly noting a distinctive property of the model when metacognitive noise is absent. The observer’s decision rule is deterministic, and was described in Section 2. If *x_lp_* < 0 the observer reports that *R* =1, and reports *R* = 2 if *x_lp_* > 0. In all cases, the observer makes the response that is most likely to be correct. Hence, (scaled or not) the log-posterior ratio at the time of the decision always favours the response made. Due to the absence of metacognitive noise, *x_lp_ = x_c_*, and hence *x_c_* at the time of decision also always favours the response made. As a result, the observer will never report a confidence of less than 50%. As soon as metacognitive noise is present, then it is no longer the case that in general *x_lp_ = x_c_*, and we need to model the difference between *x_lp_* and *x_c_*.

Returning to (12), using the relationship between *x_lp_* and *x_c_* given by (10) and using (13), we have,

(14) $$\Psi (x_{c})=\int dx_{lp}N(x_{c};x_{lp},\sigma_{m}^{2})p(R|x_{lp})N(x_{lp};\frac{E}{\theta (t_{e})},\frac{E^{2}\sigma_{\varphi }^{2}+t_{e}\sigma_{acc}^{2}}{\theta^{2}(t_{e})}).$$

Due to the observer’s deterministic decision rule, *p*(*R*|*x_lp_*) is one when *R* is consistent with the (scaled) log-posterior ratio, and zero otherwise. The product of this term and the normal distribution over *x_lp_* is a normal distribution truncated to the region where *R* and *x_lp_* are consistent. We rewrite this expression in terms of a truncated normal distribution, $TN(x_{lp};\mu_{lp},\sigma_{lp}^{2},a,b),$ where *a* and *b* indicate the points at which the distribution is truncated, and the first and second parameters, *μ_lp_* and $\sigma_{lp}^{2},$ are the mean and variance of the distribution prior to truncation, and therefore are,

(15) $$\mu_{lp}=\frac{E}{\theta (t_{e})}$$

(16) $$\sigma_{lp}^{2}=\frac{E^{2}\sigma_{\varphi }^{2}+t_{e}\sigma_{acc}^{2}}{\theta^{2}(t_{e})}.$$

This gives,

(17) $$\Psi (x_{c})=p(R|E)\int_{-\infty }^{\infty }dx_{lp}N(x_{c};x_{lp},\sigma_{m}^{2})TN(x_{lp};\mu_{lp},\sigma_{lp}^{2},a,b),$$

Where *a* = -∞, *b* = 0 for *R* = 1 or *a* = 0, *b* = ∞ for *R* = 2. These limits come from using that *p*(*R*|*x_lp_*) = 1 when *R* and *x_lp_* are consistent, and *p*(*R*|*x_lp_*) = 0 otherwise.

This integral can be performed by changing variables so that the expression becomes a convolution between a normal distribution and a truncated normal distribution. Let,

$$L=\{\begin{array}{ll} -1 & \text{if}\;a=-\infty ,b=0;\;\text{i}.e.\;R=1\\ 1 & \text{if}\;a=0,b=\infty ;\;\text{i}.e.\;R=2, \end{array}$$

then the result of the convolution is (Supplement Section E),

(18) $$\begin{array}{c} \Psi (x_{c}\text{)}=p(R|E)\frac{1}{\Phi (L\frac{\mu_{lp}}{\sigma_{lp}})}N(x_{c};\mu_{lp},\sigma_{m}^{2}+\sigma_{lp}^{2})\\ \times \Phi \text{(}L\frac{x_{c}\sigma_{lp}^{2}+\mu_{lp}\sigma_{m}^{2}}{\sigma_{m}\sigma_{lp}\sqrt{\sigma_{m}^{2}+\sigma_{lp}^{2}}}). \end{array}$$

Substituting this expression into (11), and extensively rearranging to produce an expression that is faster to evaluate numerically, gives the following final result for confidence,

(19) $$\begin{array}{r} p(C=i|R,E)=\frac{L}{\Phi \text{(}L\frac{\mu_{lp}}{\sigma_{lp}}\text{)}}[\;\;\;BvN(\frac{g}{\sqrt{1+h^{2}}},e_{i+1};\frac{-h}{\sqrt{1+h^{2}}})\\ -BvN(\frac{g}{\sqrt{1+h^{2}}},e_{i};\frac{-h}{\sqrt{1+h^{2}}})], \end{array}$$

where,

(20) $$BvN(l_{1},l_{2},\rho )=\int_{-\infty }^{l_{1}}\int_{-\infty }^{l_{2}}\frac{1}{2\pi \sqrt{1-\rho^{2}}}e^{-\frac{1}{2(1-\rho^{2})}(x^{2}+y^{2}-2xy\rho )}dxdy,$$

is the bivariate cumulative normal distribution, corresponding to a distribution with mean and covariance,

(21) $$\mu =[\begin{array}{c} 0\\ 0 \end{array}]\;\text{and}\;\Sigma =[\begin{array}{cc} 1 & \rho \\ \rho & 1 \end{array}].$$

Additionally, *g, h*, and *e_i_* denote $g=\frac{L\mu_{lp}\sqrt{\sigma_{m}^{2}+\sigma_{lp}^{2}}}{\sigma_{m}\sigma_{lp}},h=\frac{L\sigma_{lp}}{\sigma_{m}}$ and $e_{i}=\frac{Ld_{i}-\mu_{lp}}{\sqrt{\sigma_{m}^{2}+\sigma_{lp}^{2}}}.$ This integral can be numerically evaluated using standard functions.

### Free response condition

**Fig. 5:**
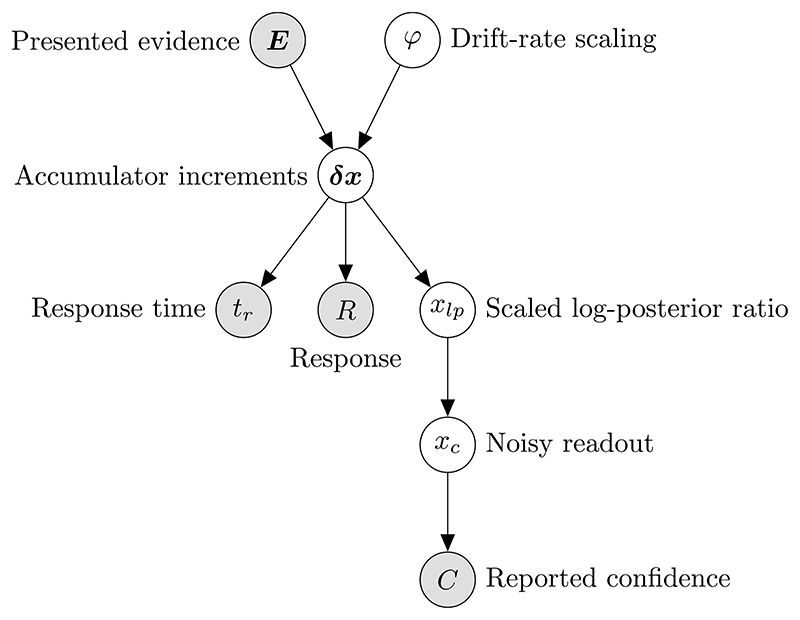
Representation of the generative model for confidence reports in the free response condition, from the perspective of the researcher. We want to infer the probability of reported confidence, using our knowledge of the evidence presented, the response given, and the response time.

In the free response condition we again want to find the probability distribution over confidence reports, but now have an additional piece of information to incorporate into our predictions, the time of the response *t_r_*. Response time will be determined by the evolution of the accumulator, and specifically, by the first time the accumulator reaches a decision threshold (Fig. 5).

Integrating *x_c_* over the region that leads to a confidence report *C* = *i* after *R* = 2, and marginalising over *x_lp_*,

(22) $$\begin{array}{ll} p(C=i|R=2,t_{r},E)= & \int_{d_{i}}^{d_{i+1}}dx_{c}\int dx_{lp}p(x_{c}|x_{lp})\\ & \times p(x_{lp}|R=2,t_{r},E). \end{array}$$

(As before, for *R* =1 we use integration limits −*d*_*i*+1_ and −*d_i_* instead.) The second distribution in this expression can be obtained by marginalising over *φ*,

(23) $$p(x_{lp}|R,t_{r},E)=\int d\varphi p(x_{lp}|\varphi ,R,t_{r},E)p(\varphi |R,t_{r},E).$$

The final term is a distribution over *φ*, the drift-rate scaling. Observers often need to calculate the distribution over drift rate, given the evidence received (Drugowitsch et al., 2012; Moran, 2015; Moreno-Bote, 2010). As researchers, we can infer the value of *φ* in a similar way.

Evidence presented immediately prior to a response but after the decision point does not contribute to the response itself, as it is still being processed (Resulaj et al., 2009). If, for an observer, the interval of stimulus in this processing pipeline is *I*, we can infer that the amount of time they spent accumulating evidence prior to a decision, *t_d_*, is *t_d_ = t_e_-I = t_r_-I*. Additionally, if an observer uses a decision threshold, *f*(*t*), to trigger responses *R* = 2, and −*f*(*t*) to trigger responses *R* = 1, then we know that at the time they made their decision *t_d_*,

(24) $$x=\{\begin{array}{ll} f(t_{d}) & \text{if}\;R=2\\ -f(t_{d}) & \text{if}\;R=1, \end{array}$$

because the time they made their decision was the time the accumulator hit the decision threshold (Moreno-Bote, 2010). Denote the value of *x* at *t_d_* by *x_d_*. Hence for known (or hypothesised) values of *I* and *f*(*t*), we can infer *t_d_* and *x_d_* from *R* and *t_r_*. Note that in this case we can also infer *R* and *t_r_* from *t_d_* and *x_d_*. Hence, these quantities are interchangeable in

(25) $$p(\varphi |R,t_{r},E)=p(\varphi |x_{d},t_{d},E).$$

The probability distribution over *φ* will depend on the entire stream of evidence up to the time of the decision (i.e., all elements of ***E*** that correspond to evidence received before a decision). This is because measurements of all evidence prior to a decision, in conjunction with *φ*, determine changes in the accumulator, which in turn determine the time of the response, and response itself (see Fig. 5). However, for the purpose of inferring *φ*, we approximate the evidence stream by its average prior to the time of the decision, *Ē*. We also approximate *p*(*φ|Ē*) ≈ *p*(*φ*). *φ* is independent of ***E***, but could depend on *Ē*, because the average evidence may be related to the time of the response, which *φ* also affects (Fig. 5). We test the effects of these approximations once the derivation is complete.

In Supplement Section F we show how these approximations, in conjunction with (25), an application of Bayes rule, and consideration of the set of paths that first cross the decision threshold at *t_d_* (Moreno-Bote, 2010), lead to the following expression,

(26) $$p(\varphi |R,t_{r},E)\propto p(\varphi )N(\varphi ;\frac{x_{d}}{\overset{-}{E}t_{d}},\frac{\sigma_{acc}^{2}}{{\overset{-}{E}}^{2}t_{d}}).$$

We have an expression for *p*(*φ*) from (5). Using this expression, (26) can be rearranged into a single normal distribution, as shown in Supplement Section F.

Returning to (23), we see that in addition to an expression for *p*(*φ*|*R,t_r_, **E***), we need an expression for *p*(*x_lp_*|*φ,R,t_r_, **E***). As discussed, from the response and response time, we can infer the time spent accumulating evidence prior to the decision, and the state of the accumulator at the time a decision was made (Fig. 2). Following a decision, evidence accumulation continues in the same manner as in the interrogation condition (Pleskac & Busemeyer, 2010), until all evidence measurements have been processed. Specifically, in the absence of decision boundaries following a decision, the distribution over changes in accumulated evidence is normal, with a mean matching the sum of presented evidence modulated by the standardised drift rate, and with a variance that grows with time. (Precisely, Eq. (73) in Supplement Section E is valid for predicting the accumulation following decision threshold crossing.)

Considering the time between the decision and the end of stimulus processing (*t_d_,t_e_*), denote the accumulation in this interval, Δ*x*, and the sum over evidence presented in this interval Δ*E = ∑_i_ δE_i_* (the summation is taken over all *i* which correspond to time steps following a decision). (Δ*E^2^* indicates (Δ*E*)^2^.) We have (see Eq. (73) in Supplement Section E),

(27) $$p(\text{Δ}x|\varphi ,E)=N(\text{Δ}x;\varphi \text{Δ}E,\sigma_{acc}^{2}I),$$

where *I* denotes the duration of the pipeline, *t_e_ - t_d_*. Using our knowledge of the location of *x* at *t_d_*, denoted *x_d_*, and that the final state of *x* is given by *x_d_* + Δ*x*, the distribution over the final state is given by,

(28) $$p(x|\varphi ,R,t_{r},E)=N(x;x_{d}+\varphi \text{Δ}E,\sigma_{acc}^{2}I)$$

Using (9) we have,

(29) $$p(x_{lp}|\varphi ,R,t_{r},E)=N(x_{lp};\frac{x_{d}+\varphi \text{Δ}E}{\theta (t_{e})},\frac{\sigma_{acc}^{2}I}{\theta^{2}(t_{e})})$$

We have now derived expressions for both the distributions in (23). In Supplement Section F we perform the integral to find,

(30) $$p(x_{lp}|R,t_{r},E)=N(x_{lp};\mu_{lf},\sigma_{lf}^{2})$$

Where,

(31) $$\mu_{lf}=\frac{1}{\theta (t_{e})}(x_{d}+\text{Δ}E\frac{\sigma_{acc}^{2}+x_{d}\sigma_{\varphi }^{2}\overset{-}{E}}{\sigma_{acc}^{2}+t_{d}\sigma_{\varphi }^{2}{\overset{-}{E}}^{2}})$$

(32) $$\sigma_{lf}^{2}=\frac{\sigma_{acc}^{2}}{\theta^{2}(t_{e})}(\frac{\text{Δ}E^{2}\sigma_{\varphi }^{2}}{\sigma_{acc}^{2}+t_{d}\sigma_{\varphi }^{2}{\overset{-}{E}}^{2}}+I)$$

Using this result in (22), and allowing for normally distributed metacognitive noise as in (10),

(33) $$\begin{array}{ll} p(C=i|R=2,t_{r},E)= & \int_{d_{i}}^{d_{i+1}}dx_{c}\int dx_{lp}p(x_{c}|x_{lp})\\ & \times p(x_{lp}|R=2,t_{r},E) \end{array}$$

(34) $$\begin{array}{l} =\int_{d_{i}}^{d_{i+1}}dx_{c}\int dx_{lp}N(x_{c};x_{lp},\sigma_{m}^{2})\\ \;\;\;\;\times N(x_{lp};\mu_{lf},\sigma_{lf}^{2}) \end{array}$$

(35) $$=\int_{d_{i}}^{d_{i+1}}dx_{c}N(x_{c};\mu_{lf},\sigma_{lf}^{2}+\sigma_{m}^{2}).$$

This expression applies for *R* = 2. For the case of *R* = 1 the limits change to *−d_i+1_* and *−d_i_*.

Thus, we have derived expressions for the probability of confidence reports falling into each ordinal bin, given the trial-by-trial response, response time and stimulus. We have produced such expressions for both interrogation (19) and free response (35) decision tasks. By allowing for the possibility of various additional features in the underlying decision and confidence model, the derivations build on previous work, as set out in further detail in the discussion below. Eqs. (19) and (35) provide approximate expressions for confidence that only need evaluating once per trial, with the aim of supporting feasible trial-by-trial modelling even with stimuli that fluctuate stochastically within each trial.

### Testing the approximations

We made several approximations in the derivations above, so it is important to check that our predictions for confidence closely match confidence reports, when these are simulated. We simulated the diffusion process using small time steps, and produced confidence reports in accordance with the model (see (9), (10) and Fig. 2; for details of the simulations see Supplement Section G). We then took each trial and computed predictions for the probability of each confidence report using the derived expressions, before randomly drawing a confidence report in accordance with the probability assigned to it. This allowed us to plot confidence simulated from the model, and confidence reports that match the derived predictions.

**Fig. 6:**
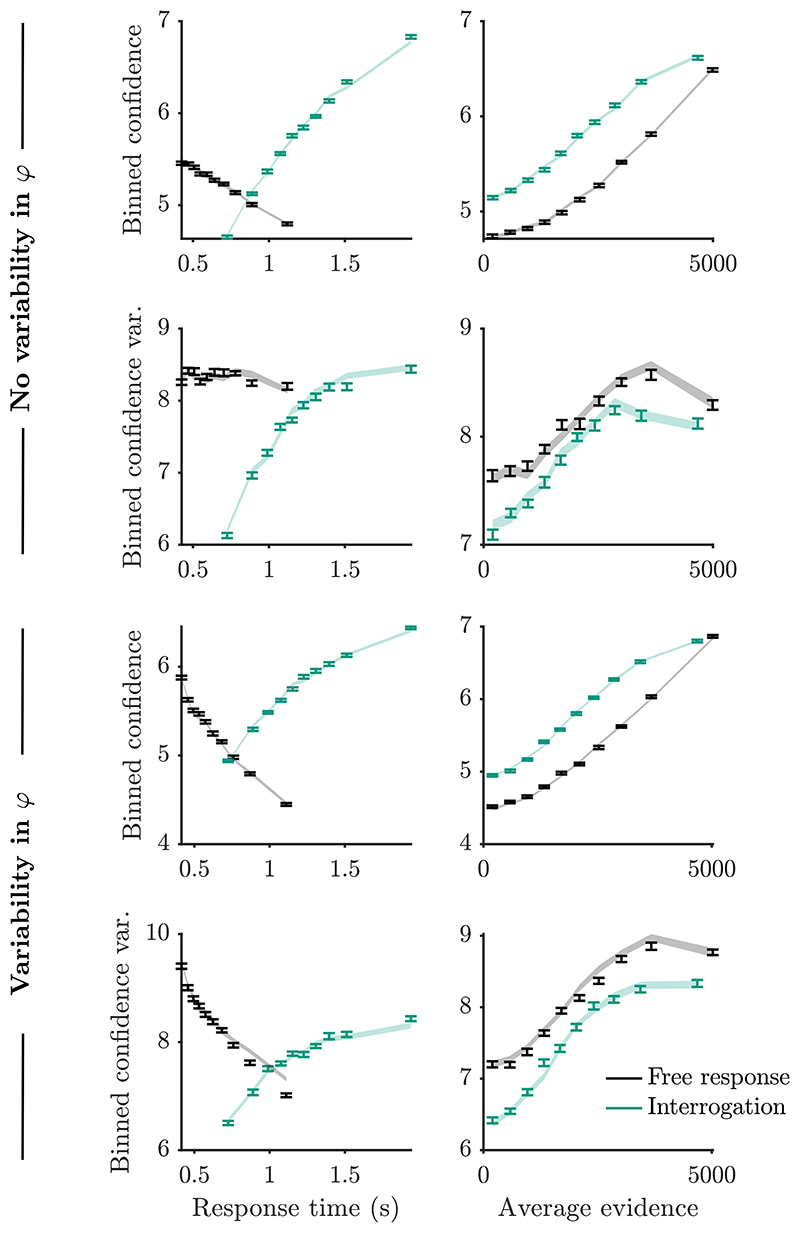
Mean and variance of binned confidence, produced via simulation of the model (error bars), and through the derived predictions (shading). “Average evidence” here refers to the absolute value of the following quantity: The difference in dots summed over all presented frames, divided by the duration of stimulus presentation. Details of the simulation and plotting are provided in Supplement Section G. Predictions matched the simulation closely. There were some signs that approximations used to derive the predictions lead to a small overestimation of variability in confidence when there is variability in drift-rate scaling (*φ*).

Fig. 6 shows simulations of confidence using the model (error bars), and confidence based on the derived predictions (error shading). No additional approximations were made in deriving confidence predictions in the interrogation condition. Consistent with this, simulated confidence and the variance of simulated confidence closely match predicted confidence and predicted confidence variance, as functions of response time and unsigned average evidence over the entire-stimulus, both with and without variability in drift-rate scaling.

For the free response derivations, we approximated evidence prior to a decision by its average, for the purpose of estimating the driftrate scaling, and approximated the drift-rate scaling as independent of the average evidence prior to a decision. These approximations are only relevant when the drift-rate scaling is variable. Consistent with this, simulated and predicted confidence closely match in plots corresponding to no variability in drift-rate scaling *φ* (Fig. 6). When variability in drift-rate scaling is present, we can see that the approximations introduce some small discrepancies between simulations and predictions. For example, the predictions appear to overestimate the variability in confidence reports in trials with long response times. For completeness, we note that there were similarly small discrepancies in an observer who used an alternative to Bayesian confidence (Supplement Section H). Nevertheless, we should always be mindful of the fact that an approximation that works for one model or set of parameters, may not work so well for another model or set of parameters.

## 4 Discussion

Using the normative frameworks of the DDM for decision making and a Bayesian readout for confidence, we derived predictions for the probability distribution over confidence reports, given the response, response time, and stimulus presented on a trial. We considered both the typical case, where response time is under the control of the participant (free response), and the less common case in which the observer has to respond at a particular time (interrogation). In the free response case, where the observer must set decision thresholds to trigger a response, we allow for the use of decision thresholds of arbitrary shape. The results, summarised in Table 3, build on the work of Moreno-Bote (2010) by including features that are important in the construction of confidence. Specifically, the derivations account for accumulation of pipeline evidence (Moran et al., 2015), the effect of drift rate variability on pipeline evidence (Pleskac & Busemeyer, 2010), and metacognitive noise (Maniscalco & Lau, 2012, 2016).

Importantly, the derivations cover not only static stimuli but also stochastically fluctuating stimuli that generate normally distributed fluctuations in evidence signal each frame. The derived expressions only require one evaluation per trial, in contrast to previous approaches that could handle dynamic stimuli but based on evaluation of some function at every time step prior to a decision (e.g. Chang & Cooper, 1970; Smith, 2000; Voss & Voss, 2008; Zylberberg et al., 2018). Reducing computational cost is crucial for making trial-by-trial modelling of stochastically fluctuating stimuli feasible. Trial-by-trial modelling may provide stronger constraints when fitting models than predictions made for large groups of trials at once (Park et al., 2016), which has until now been the standard approach (see Section 1). Computationally cheap predictions may also allow us to use techniques which require predictions to be evaluated many times, such as cross-validation and Markov chain Monte Carlo (MCMC; Bishop, 2006). A key insight behind our derivations is that it can be much more tractable to model the probability distribution over confidence reports than it is to derive computationally cheap expressions for the decisions (and associated response times) to which the confidence reports relate. This is because in the build up to a confidence report, and specifically after the decision threshold has been crossed, the evolving state of the accumulator follows a normal distribution. In contrast, in the lead up to a decision, the accumulation – constrained to lie at or below a decision boundary – is inherently non-normal.

One attractive aspect of the derivations is that – should methods be developed that facilitate efficient trial-by-trial computation of the probability of responses and response times themselves – these can be straightforwardly incorporated, allowing us to perform simultaneous trial-by-trial modelling of decisions, response times, and confidence. For the latter, we need to know the joint probability distribution over decisions, response times, and confidence, given the stimulus presented on a specific trial. This joint probability can be decomposed as follows,

(36) $$p(R,t_{r},C|E)=p(C|R,t_{r},E)p(R,t_{R}|E).$$

We have produced computationally cheap expressions for the first term on the right hand side of this equation. If future research produces computationally cheap expressions for the second term, these expressions can be combined through simple multiplication. We have focused on modelling confidence to make trial-by-trial modelling using existing methods feasible. Nevertheless, our derivations can be easily combined with future work, should new methods be developed, permitting even more precise and constrained model fitting, model comparison, and parameter estimates.

For readability, we have kept the dependence of the predictions for confidence on model parameters implicit (Section 3), but it will be by adjusting these parameters that we can fit the model described to data (Table 2). Additionally, by constraining parameters to certain values we can construct model variants for model comparison. For example, we could ask whether metacognitive noise is an important source of variability by comparing a model in which we fit all parameters, to a model in which the standard deviation of metacognitive noise is set to zero. There is special flexibility with the decision threshold, because the modeller can choose its shape and how to parameterise it. Using these derivations we have recently compared a variety of models of confidence, including models in which the decision threshold is flat, and models with a decreasing decision threshold (Calder-Travis et al., 2020).

At the outset we noted that simple expressions may also provide additional insights into the mechanisms responsible for confidence. For example, such expressions may elucidate exactly how we expect different variables to interact to generate confidence, thereby helping us to understand and relate the various patterns that have been observed in confidence data. Consider a situation in which observers report their confidence on a very fine-grained scale in a free response task. In the simple case where the observer scales their readout so that it matches the log-posterior ratio, *x_lp_/K*, the most likely confidence report is (using Eq. (31)) given by the following,

(37) $$\begin{array}{r} \frac{1}{K\theta (t_{e})}(x_{d}+\text{Δ}E\frac{\sigma_{acc}^{2}+x_{d}\sigma_{\varphi }^{2}\overset{-}{E}}{\sigma_{acc}^{2}+t_{d}\sigma_{\varphi }^{2}{\overset{-}{E}}^{2}})=\frac{2t_{f}\text{Δ}\mu }{t_{f}^{2}\sigma_{acc}^{2}+t_{f}\sigma_{E}^{2}+t_{e}\text{Δ}\mu^{2}\sigma_{\varphi }^{2}}\\ \times (x_{d}+\text{Δ}E\frac{\sigma_{acc}^{2}+x_{d}\sigma_{\varphi }^{2}\overset{-}{E}}{\sigma_{acc}^{2}+t_{d}\sigma_{\varphi }^{2}{\overset{-}{E}}^{2}}). \end{array}$$

Breaking this expression down, we see that the prefactor multiplying the term in brackets features various sources of variability in the denominator. This factor generates a highly intuitive relationship: When variability increases (and the observer detects this increase in variability) confidence should tend to decrease. Beyond this, the second half of (37), in parentheses, represents a derivation and expression of key principles of the 2DSD model of confidence introduced by Pleskac and Busemeyer (2010). In particular, assuming no variability in drift-rate scaling, the only evidence used for predicting confidence is evidence from the processing pipeline. This is because if drift-rate scaling variability, *σ_φ_*, is zero, the second term within the parentheses,

(38) $$\text{Δ}E\frac{\sigma_{acc}^{2}+x_{d}\sigma_{\varphi }^{2}\overset{-}{E}}{\sigma_{acc}^{2}+t_{d}\sigma_{\varphi }^{2}{\overset{-}{E}}^{2}},$$

simply reduces to Δ*E*. It seems counterintuitive that the evidence on which the decision was based adds nothing to our prediction for confidence. This occurs because, at the time of the decision, we know that the state of the accumulator is at the decision boundary corresponding to the response made (Fig. 2; Pleskac & Busemeyer, 2010; Yu et al., 2015). Given knowledge of the state of the accumulator at the time of the decision, we do not need to know the evidence presented up to this point.

Drift rate variability adds nuance to this relationship, via the fraction multiplying Δ*E* in (38). This term provides a mathematical description of the fact that, if strong evidence has been gathered by the time of the decision, relative to the time spent deliberating, the drift-rate scaling is likely to be high (Moreno-Bote, 2010), and pipeline evidence will have a big impact on decision confidence (Pleskac & Busemeyer, 2010). On the other hand, if at the time of decision, little evidence has been gathered relative to the time spent deliberating, evidence is accumulating slowly, suggesting a low drift-rate scaling. In turn, this suggests that pipeline evidence will be processed poorly and will have a small effect on confidence. This is why the fraction contains decision time in the denominator, reducing the effect of pipeline evidence, and the height of the threshold at decision time is in the numerator, increasing the effect of pipeline evidence. We are not the first to describe this effect of drift rate variability; it was a central idea in the model for confidence proposed by Pleskac and Busemeyer (2010). Our contribution is to derive an expression for this effect. Moreover, the expression in (37) goes beyond the 2DSD model to include effects of time on confidence stemming from the Bayesian confidence readout used (Moran, 2015; Moreno-Bote, 2010), that are present even in the absence of continued evidence accumulation following a decision, i.e., when Δ*E* is zero. Even in this case, the total time spent processing the stimulus, *t_e_*, still appears in the denominator of the prefactor in (37), and will therefore reduce confidence, consistent with previous findings (Kiani et al., 2014; Murdock & Dufty, 1972).

Beyond formalising our ideas about the relationship between confidence and other variables, a further insight from our expressions for confidence is the integration of empirical findings that previously appeared difficult to explain. In particular, there are inconsistent findings regarding the relationship between confidence and signal strength on error trials. Sanders et al. (2016) found confidence on error trials decreased as signal strength increased, which has been taken as a distinguishing feature of confidence in some studies (Kepecs & Mainen, 2012). However, Hellmann et al. (2023) and Kiani et al. (2014) found that confidence on error trials increased with signal strength. In these studies, participants simultaneously reported their decisions and confidence, and Kiani et al. (2014) suggested that this design choice may have been key to the pattern they observed (see also Desender et al., 2020; Khalvati et al., 2020).

Our derivations support this suggestion. To see why, we look again at our expression for most likely confidence, (37), but now consider the situation in which *ΛE* = 0 (as would be the case if choice and confidence are reported simultaneously). We have,

(39) $$\frac{2t_{f}\text{Δ}\mu }{t_{f}^{2}\sigma_{acc}^{2}+t_{f}\sigma_{E}^{2}+t_{e}\text{Δ}\mu^{2}\sigma_{\varphi }^{2}}x_{d}.$$

In the case of a flat decision threshold (and, hence, constant *x_d_*), the only variable that changes between trials is *t_e_*, the duration of evidence presentation, which is the same as response time in the free response task. Noting that this term appears in the denominator of the expression, that greater signal strength will lead to faster responses, and that at each level of signal strength the response times for correct and error trials will on average be identical (in the absence of drift-rate variability; Ratcliff & McKoon, 2008; Ratcliff & Rouder, 1998; Shadlen et al., 2006), we therefore predict that the most likely confidence report will also be higher on both correct and error trials, due to the lower *t_e_*, when signal strength is greater. (The pattern of response times in Experiment 1 of Hellmann et al., 2023 is not consistent with this account, but we note that all models struggled to account for the change in response times with signal strength in this experiment, and speculate that it may be important to take into account perceptual integration when stimulus masking is used.) A different prediction follows when the observer has time to process pipeline evidence. The processing pipeline contains a considerable amount of information from the stimulus (approximately 400 ms of the stimulus prior to response; Ratcliff & McKoon, 2008; Resulaj et al., 2009). On error trials, this evidence will tend to favour the alternative (correct) option, decreasing confidence, and this effect will be stronger when signal strength is high. The implication is that confidence will decrease as signal strength increases.

**Fig. 7:**
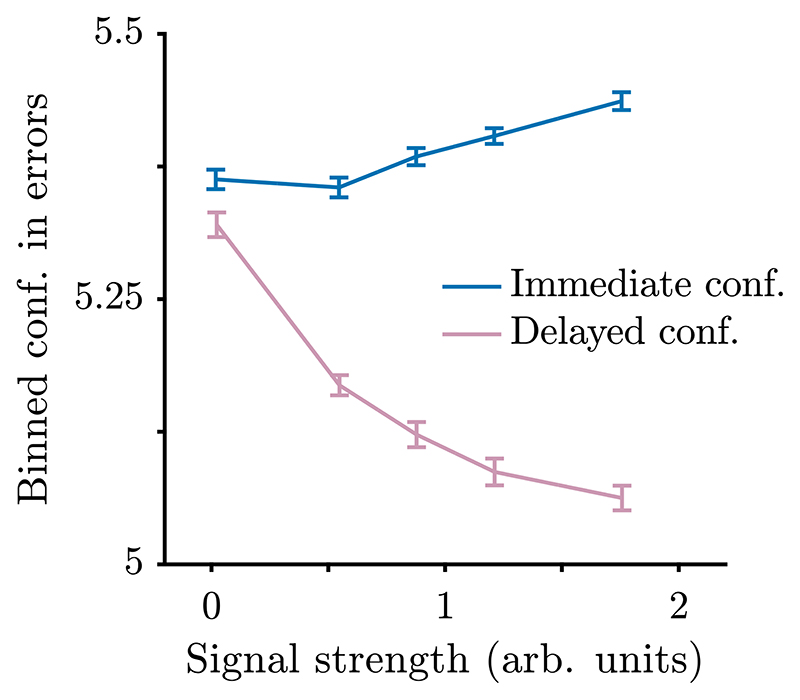
Confidence on free response trials that led to errors, as a function of signal strength. When confidence is reported immediately, and therefore does not reflect evidence measurements in the processing pipeline, confidence on error trials increases with signal strength (*t*(39) = 7.1, *p* = 1.4× 10^-8^). On the other hand, when confidence reports are made after a decision, and include 100 ms of evidence measurements from the processing pipeline, confidence on error trials decreases with signal strength (*t*(39) = -17, *p* = 7.7 × 10^-20^). Variability in signal strength was generated by using a non-zero value for drift-rate variability. To perform the statistical tests, for each participant and condition, we calculated the correlation coefficient between the signal strength and binned confidence on error trials. We then compared the correlation coefficients to zero across participants. Simulation and plotting details in Supplement Section G.

To test the intuitions gained from studying the equations, we simulated response times, decisions, and confidence reports under two different free response conditions. In one condition, we simulated the task designed by Kiani et al. (2014), setting pipeline evidence to zero. In the other condition, we simulated a processing pipeline containing (a conservative) 100 ms of the stimulus prior to response (see Supplement Section G for details of the simulations). Confidence in errors increased with signal strength when decisions and confidence reports were simultaneous, whereas confidence in errors decreased with signal strength when observers received pipeline evidence (Fig. 7).

In this way, the expressions we derive can provide new insights as well as formalise key intuitions about confidence in tractable expressions for use in future modelling efforts. We should, however, note some important limitations to our approach. First, regarding the scope of the derivations, we have been concerned only with the DDM framework and with a specific class of stimuli. We focus on the DDM as a particularly interesting case due to the normative properties of the diffusion mechanism (see Section 1). Nevertheless, a family of alternatives to the DDM have been studied that assume not just one accumulator, but two (Bogacz et al., 2006; Moreno-Bote, 2010), which may be more or less anti-correlated with each other. It may be possible to use recently derived expressions for the state of the second accumulator at decision time, to extend the approach developed here (Shan et al., 2019), but we leave this for future research.

Likewise, we leave for future research whether it is possible to extend the derivations to more general dynamic stimuli, rather than just those with normally distributed evidence fluctuations and for which the effects of perceptual integration can plausibly be ignored (see Section 2). In principle, any stimulus for which we can find the optimal observer’s decision and confidence rules could be modelled using the approach we have described. One of the most common stimuli, the random dot motion stimulus, contains dots that move randomly over the course of a trial, creating random fluctuations in evidence for the prevailing motion direction (Kiani et al., 2008; Pilly & Seitz, 2009). If it was possible to characterise the nature of these fluctuations, and derive the optimal observer’s decision and confidence rule, a very large quantity of data could be analysed on a trial-by-trial basis using the approach set out here. A specific challenge that would need to be overcome in the case of the random dot motion task would be taking into account the effects of perceptual integration, which are likely substantial for this task (Smith & Lilburn, 2020; Watamaniuk & Sekuler, 1992). Perceptual integration will complicate the optimal observer’s computation for confidence, because evidence received at different time points will no longer be (conditionally) independent (given the standardised drift-rate). A Bayesian observer will take into account these additional, short-term correlations, and future theoretical work will be needed to determine the Bayesian confidence computation under such conditions.

A further direction in which the derivations could be generalised to apply to more stimuli would be to consider a greater range of priors over mean evidence strength (given by *μ*, see Eq. (2)). The derivations already cover stimulus frame-to-frame stochastic fluctuations around the mean evidence strength, and trial-to-trial variability in how well the stimulus evidence is processed (given by the drift-rate scaling, *φ*). This creates a situation in which observers are aiming to infer from which of two overlapping normal distributions the average rate of evidence accumulation is drawn (see Eqs. (6) and (7)). Distinguishing between two classes of stimuli, whose features possibly overlap (e.g., Adler & Ma, 2018), is a common task for animals and humans (e.g., determining whether a distant shape is a predator or not). A different situation that has also been considered is the task of determining whether the average rate of evidence accumulation is positive or negative (e.g., Moreno-Bote, 2010), corresponding to the different task of directly comparing a stimulus feature to a reference value (e.g., determining whether an animal is running faster or slower than a specific speed). The approach presented here could also be adapted to this other form of task. The main change would be in terms of how the observer converts the accumulated evidence into an estimate of confidence: The precise form of the normative transformation will be different, reflecting the different goal of the observer (Moreno-Bote, 2010).

A related limitation is that we have only considered a specific kind of confidence report – a Bayesian readout of the probability of being correct – and have assumed that the observer holds approximately true beliefs about the generative process responsible for their evidence measurements. It is important to note that observers can behave as if they hold a true generative model, and match the behaviour of a Bayesian observer, simply by learning through association the mapping from accumulator state and time to probability correct (Kiani & Shadlen, 2009; Ma & Jazayeri, 2014). Therefore, human confidence reports may be well described by the equations derived above, even if humans are not actually performing Bayesian computations, if feedback is provided and participants have sufficient time to learn to report their confidence appropriately. In addition, the derived equations could be adapted to cover cases where the generative model held by the observer does not perfectly match the true generative model (as in Calder-Travis et al., 2020 for example). It will be through empirical investigation that we determine if and when confidence reports are Bayesian. In providing new computationally cheap expressions for Bayesian confidence, we hope to support empirical work that aims to understand the complicated and mixed pattern of results relevant to this topic (e.g. Adler & Ma, 2018; Caziot & Mamassian, 2021; Desender et al., 2020; Geurts et al., 2022; Khalvati et al., 2020; Kvam & Pleskac, 2016; Li & Ma, 2020; Meyniel et al., 2015; Peters et al., 2017; Sanders et al., 2016).

A second set of limitations concern the applicability of our derivations to modelling. In particular, we only make predictions for confidence, not for response times and decisions, and we have not yet accounted for lapses. As detailed above, our choice to derive expressions for confidence alone was a deliberate one, made in order to avoid the difficulties in deriving computationally cheap expressions for responses and response times (see Section 1). Additionally, when fitting confidence reports, we will still model the decision mechanism, in the sense that we will generate estimates for the parameters of this mechanism. Using these parameters we will be able to make predictions for decisions and response times. This will allow us to examine whether a model that fits well to confidence nevertheless generates implausible response and response time data, and will provide an additional check of the model and its assumptions. Regarding lapses, it would be tricky to directly model lapses that affect the response produced (Adler & Ma, 2018; Ratcliff & Tuerlinckx, 2002), because we do not have expressions for the probability distribution over decisions and response times generated by the non-lapse diffusion process. (For similar reasons, it would be difficult to incorporate the idea of variability in the start point of the accumulator, and variability in the duration of the pipeline (Ratcliff & McKoon, 2008; Ratcliff & Tuerlinckx, 2002).) However, it would be straightforward to include a lapse rate parameter that describes some probability that a random confidence report is given.

Notwithstanding the limitations discussed, we believe these derivations will prove useful for the two purposes described at the outset: Supporting deeper insight into the confidence of an important class of observers, and supporting trial-by-trial modelling. We have seen in this discussion that the derived expressions offer the potential to directly explore and understand the relationship between confidence and other variables. This level of explanatory power may be difficult to gain even after running simulations using a wide range of parameter values. The expressions found only require evaluating once per trial, making trial-by-trial modelling of stochastically fluctuating stimuli more feasible. As a consequence, we hope these results will support efforts to develop models which make ever more precise and sophisticated predictions for behaviour.

## Supplementary Material

Appendix

## Figures and Tables

**Table 1:** Symbols used in the derivations, along with key abbreviations.

Symbol	Meaning
*s*	Stimulus (1 or 2)
*R*	Response (1 or 2)
*C*	Confidence
*μ*	Difference between mean stimulus evidence for the two options
Δ*μ*	Absolute value of the difference between the two means
*E_i_*	Difference in evidence presented in stimulus frame *i*
*δE_i_*	Difference in evidence presented in time step *i*.
	Note, *E_i_* = ∑_*i*_ *δE_j_* (sum taken over time steps in a frame)
*E*	Sum over all *E_i_*
***E***	Vector of all *E_i_*
*Ē*	Average difference in evidence between the two options, prior to decision
Δ*E*	Sum over *δE_i_* for time steps following the decision. Note Δ*E*^2^ indicates (Δ*E*)^2^.
*φ*	Drift-rate scaling
*δx_i_*	Difference between the evidence measurements in time step *i*
***δx***	Vector containing *δx_i_* for every *i*
*x*	Accumulated difference in measurements. ∑_*i*_ *δx_i_*
*x_d_*	The value of *x* at *t_d_* (i.e. height of relevant decision threshold at this time)
Δ*x*	The change in *x* following a decision
*x_lp_*	Scaled log-posterior ratio
*x_c_*	Noisy measurement of *x_lp_* which determines confidence
*d_i_*	Boundaries on *x_c_* which separate confidence reports falling into different bins
*σ_E_*	Standard deviation of evidence in a frame
*σ_acc_*	Standard deviation of accumulator noise
*σ_φ_*	Standard deviation of *φ*
*σ_m_*	Standard deviation of metacognitive noise
*t_f_*	Duration of a frame
*t_e_*	Duration of evidence presentation
*t_d_*	Duration from onset of accumulation to first crossing of a decision threshold
*t_r_*	Response time
	(In free response condition *t_r_ = t_e_ = t_d_* + *I*; in interrogation condition *t_r_ = t_e_* +*I*)
*I*	The duration of the evidence pipeline
Symbol	Abbreviates
*ν*	*φμ*/*t_f_*
*ν*_0_	Δ*μ*/*t_f_*
*σ_ν_*	*ν*_0_ *σ_φ_*
*s^2^*	$\sigma_{acc}^{2}+(\sigma_{E}^{2}/t_{f})$
*θ*(*t*)	$\left(s^{2}+t\sigma_{\nu }^{2})/(s^{2}+\sigma_{\nu }^{2}\right)$
*K*	$(s^{2}+\sigma_{\nu }^{2})/2\nu_{0}$

**Table 2:** Model parameters for fitting to data. The predictions for confidence depend on these parameters, but this dependence is kept implicit throughout for readability (i.e., a symbol for the set of parameters is not explicitly included when writing out conditional probabilities). The table states “Parameter/feature” because *f*(*t_d_*) is not a parameter. *f*(*t_d_*) describes how the shape of the decision threshold changes over time. The modeller can parameterise this function as they wish. For example, they could use a flat threshold and simply fit threshold height, or they could use a complicated curved threshold with several parameters.

Parameter/feature		Free response	Interrogation
Accumulator noise	*σ_acc_*	✓	✓
Drift-rate variability	*σ_φ_*	✓	✓
Metacognitive noise	*σ_m_*	✓	✓
Confidence boundaries	*d_i_* (for all *i*)	✓	✓
Duration of evidence pipeline	*I*	✓	–
Decision threshold shape	*f*(*t_d_*)	✓	–

**Table 3:** Summary of the derived expressions for confidence. In the interrogation condition expression *L* = 1 if *R* = 2 and *L* = -1 if *R* = 1. The free response expression applies for *R* = 2, while for *R* = 1 the integral limits change to −*d*_*i*+1_ and −*d_t_*. See Table 1 for further symbol definitions..

Condition	Expression for confidence
Interrogation	$\begin{array}{lll} p(C=i|R,E) & = & \frac{L}{\Phi \text{(}L\frac{\mu_{lp}}{\sigma_{lp}}\text{)}}[BvN(\frac{g}{\sqrt{1+h^{2}}},e_{i+1};\frac{-h}{\sqrt{1+h^{2}}})\\ & & -BvN(\frac{g}{\sqrt{1+h^{2}}},e_{i};\frac{-h}{\sqrt{1+h^{2}}})].\; \end{array}$
	Where,
	$\begin{array}{lll} BvN(l_{1},l_{2},\rho ) & = & \int_{-\infty }^{l_{1}}\int_{-\infty }^{l_{2}}\frac{1}{2\pi \sqrt{1-\rho^{2}}}e^{-\frac{1}{2(1-\rho^{2})}(x^{2}+y^{2}-2xy\rho )}dxdy\\ \;\;\;g & = & \frac{L\mu_{lp}\sqrt{\sigma_{m}^{2}+\sigma_{lp}^{2}}}{\sigma_{m}\sigma_{lp}}\\ \;\;\;h & = & \frac{L\sigma_{lp}}{\sigma_{m}}\\ \;e_{i} & = & \frac{Ld_{i}-\mu_{lp}}{\sqrt{\sigma_{m}^{2}+\sigma_{lp}^{2}}}\\ \;\;\;\mu_{lp} & = & \frac{E}{\theta (t_{e})}\\ \;\;\;\sigma_{lp}^{2} & = & \frac{E^{2}\sigma_{\varphi }^{2}+t_{e}\sigma_{occ}^{2}}{\theta^{2}(t_{e})} \end{array}$
Free response	$p(C=i|R=2,t_{r},E)=\int_{d_{i}}^{d_{i+1}}dx_{c}N(x_{c};\mu_{lf},\sigma_{lf}^{2}+\sigma_{m}^{2})$
	Where,
	$\begin{array}{l} \mu_{lf}=\frac{1}{\theta (t_{e})}(x_{d}+\text{Δ}E\frac{\sigma_{acc}^{2}+x_{d}\sigma_{\varphi }^{2}\overset{-}{E}}{\sigma_{acc}^{2}+t_{d}\sigma_{\varphi }^{2}{\overset{-}{E}}^{2}})\\ \sigma_{lf}^{2}=\frac{\sigma_{occ}^{2}}{\theta^{2}(t_{e})}(\frac{\text{Δ}E^{2}\sigma_{\varphi }^{2}}{\sigma_{occ}^{2}+t_{d}\sigma_{\varphi }^{2}{\overset{-}{E}}^{2}}+I) \end{array}$

## Data Availability

All code written for the study will be made publicly available upon publication, and will be accessible through https://doi.org/10.17605/OSF.IO/TK3VP.
