# Supplementary material for: Expressions for Bayesian confidence of drift diffusion observers in fluctuating stimuli tasks
Source: J Math Psychol. Author manuscript; Available in PMC 2024 Jan 5. (PMC7615478; doi:10.1016/j.jmp.2023.102815)
Supplement: Appendix [file EMS192935-supplement-Appendix.pdf]

# Supplementary information

Numbered sections refer to the numbered sections in the main text. Equation and figure numbering continues from the main text (e.g. equation numbers below 40 refer to equations in the main text).

# A Product of normal distributions

We will repeatedly encounter a situation where we are interested in the probability distribution over $z$ in the following equation,

$$p(z) \propto \int N(y; z, \sigma_A^2) N(y; \mu, \sigma_B^2) dy \,, \tag{40}$$

where $N$ denotes the normal distribution. The product of two normal distributions is a scaled normal distribution (over $y$; Bromiley, 2014). The scaling is given by,

$$S = N(z; \mu, \sigma_A^2 + \sigma_B^2) \,. \tag{41}$$

Once we have integrated over $y$, only the scaling is left, as the normal distribution over $y$ integrates to one. Hence,

$$p(z) = N(z; \mu, \sigma_A^2 + \sigma_B^2) \,. \tag{42}$$

# B The observer

This supplement section describes in more detail the observer that was outlined in Section 2. In particular, working from the mathematical description of the task in Section 2 we first derive the inference problem that the observer solves. We then find the computations used by a Bayesian observer to determine their choices and confidence.

Although observers could in principle leverage knowledge that evidence is constant throughout each frame, we assume that observers ignore this additional structure. This will be a valid approximation when evidence frames used in an experiment are sufficiently short. In this case, the approximation will lead to little discrepancy between the observer's estimate of the probability of being correct, and the true probability. We test this claim below, once we have derived an expression for the probability of being correct (Fig. 8, and equation 54). Instead of accounting for the constant evidence over the course of a frame, we assume that observers treat the fraction of evidence received in a short time step, $j$, as generated according to,

$$p(\delta E_j|\mu) = N(\delta E_j; \mu\frac{\delta t}{t_f}, \sigma_E^2\frac{\delta t}{t_f})\,. \tag{43}$$

Importantly, the observer incorrectly assumes that the $\delta E_j$ at different time steps, $j$, are independent (i.e., the observer ignores the fact that evidence is constant through all time steps in a single frame).

We assume that observers correctly model the effect of internal noise (accumulator noise, and drift-rate scaling variability). We address the plausibility of this assumption in Section 4. Hence observers infer that increments in the accumulator are related to the underlying signal as follows,

$$\begin{aligned} p(\delta x_j|\mu,\varphi) &= \int d(\delta E_j)\ p(\delta x_j|\delta E_j,\varphi)p(\delta E_j|\mu) && (44)\\ &= \int d(\delta E_j)\ N(\delta x_j;\delta E_j\varphi,\sigma_{acc}^2\delta t)N(\delta E_j;\mu\frac{\delta t}{t_f},\sigma_E^2\frac{\delta t}{t_f}) && (45)\\ &= N(\delta x_j;\mu\varphi\frac{\delta t}{t_f},\delta t(\sigma_{acc}^2+\varphi^2\frac{\sigma_E^2}{t_f}))\,. && (46)\end{aligned}$$

To reach the final line we rearranged and applied the result in Supplement Section A. In words, this equation states that evidence measurements have a mean that matches the underlying stimulus signal multiplied by the drift-rate scaling, but are variable due to both internal accumulator noise and the fact that evidence in the stimulus is itself variable. It is interesting to note that the drift-rate scaling, $\varphi$, not only affects the mean increment, but also the variability of increments, through the term $\varphi^2\sigma_E^2/t_f$. This effect arises because high drift-rate scaling increases the effect of the stimulus on the accumulation, increasing the effects of both the stimulus mean and variability in the stimulus. We assume that participants ignore this difference between stimulus variability and internal variability (which is not affected by drift-rate scaling). Hence we assume that observers believe evidence measurements are corrupted by the same amount of variability regardless of the level of drift-rate scaling:

$$p(\delta x_j|\mu,\varphi) = N(\delta x_j;\mu\varphi\frac{\delta t}{t_f},\delta t(\sigma_{acc}^2+\frac{\sigma_E^2}{t_f}))\,. \tag{47}$$

We test the effect of this assumption on the calibration of confidence below (Fig. 8).

To simplify these expressions we change variables to $\nu = \frac{1}{t_f}\varphi\mu$, and $s^2 = \sigma_{acc}^2 + \frac{\sigma_E^2}{t_f}$. This gives,

$$p(\delta x_i|\nu) = N(\delta x_i;\nu\delta t, s^2\delta t)\,. \tag{48}$$

We can compute the probability distribution over $\nu$ given $S$ using (2) and (5), giving,

$$p(\nu|S=1) = N(\nu;-\frac{\Delta\mu}{t_f},\frac{\Delta\mu^2}{t_f^2}\sigma_\varphi^2) = N(\nu;-\nu_0,\sigma_\nu^2) \tag{49}$$

$$p(\nu|S=2) = N(\nu;\frac{\Delta\mu}{t_f},\frac{\Delta\mu^2}{t_f^2}\sigma_\varphi^2) = N(\nu;\nu_0,\sigma_\nu^2)\,, \tag{50}$$

where $\nu_0 = \frac{\Delta\mu}{t_f}$, and $\sigma_\nu = \frac{\Delta\mu}{t_f}\sigma_\varphi$.

Having specified the properties of the task and the beliefs of the observer, we can infer the rule used by a Bayesian observer to translate all evidence measurements gathered so far into a decision and confidence. We denote the vector of all evidence measurements, $\delta x_1, \delta x_2, ..., \delta x_N$ as $\boldsymbol{\delta x}$. Inferring the posterior probability over $S$, given $\boldsymbol{\delta x}$, in the environment described by (6), (7), and (8), is an inference problem that has been considered before. Moran (2015), using a result from Drugowitsch et al. (2012), derived the posterior probability over the two options $S = 1$ and $S = 2$. We also provide a derivation in Supplement Section C, and simply state the result in this supplement section. Using Bayes rule with all evidence samples, the log-posterior ratio is given by (Supplement Section C; Moran, 2015),

$$\log \frac{p(S=2|\boldsymbol{\delta x})}{p(S=1|\boldsymbol{\delta x})} = \frac{2x\nu_0}{s^2 + t\sigma_\nu^2}\,, \tag{51}$$

where $x = \sum_{i=1}^{N} \delta x_i$, and $t$ is the time spent accumulating evidence. We will often work with a scaled version of the log-posterior ratio,

$$x_{lp} = K \log \frac{p(S=2|\boldsymbol{\delta x})}{p(S=1|\boldsymbol{\delta x})} = x\frac{s^2 + \sigma_\nu^2}{s^2 + t\sigma_\nu^2} = \frac{x}{\theta(t)}\,, \tag{52}$$

where $x_{lp}$ denotes the scaled version of the log-posterior ratio. $\theta()$ provides an abbreviation for the purpose of making future equations less cluttered. $K$ is the scaling constant and is equal to,

$$K = \frac{s^2 + \sigma_\nu^2}{2\nu_0}\,. \tag{53}$$

Note that the log-posterior ratio without any scaling is given by $x_{lp}/K$.

The observer can use the (scaled) log-posterior ratio in the computation of confidence, as it is monotonically related to the probability they are correct through,

$$p(\text{correct}|\boldsymbol{\delta x}) = p(S=1|\boldsymbol{\delta x}) = \frac{1}{1 + e^{x_{lp}/K}} = \frac{1}{1 + e^{\frac{1}{K}\frac{x}{\theta(t_e)}}}\,, \tag{54}$$

if the observer reports $R = 1$. (This expression can be found by rearranging $p(S=1|\boldsymbol{\delta x}) + p(S=2|\boldsymbol{\delta x}) = 1$.) A similar expression holds for $R = 2$, the only difference being that $x$ is replaced with $-x$, and $x_{lp}$ with $-x_{lp}$.

## Testing observer model approximations

Above, we assumed that observers approximate the true generative model for the evidence measurements. Specifically we assumed, (a) observers ignore the fact that evidence is constant within each frame, and (b) observers ignore the increased effect of variability in the stimulus, when the drift-rate scaling is high, and the decreased effect when the drift-rate scaling is low. While the assumptions may be plausible, it is also plausible that, if given feedback, observers could learn to closely approximate the mapping from $x$ and $t_e$ to probability correct (Kiani & Shadlen, 2009). To adequately capture this situation we need to ensure that, under our assumptions about the observer, confidence remains closely related to performance.

To test the two approximations, we simulated the diffusion process using small time steps. At each time step the accumulator increment, $\delta x_i$, was determined by drawing a value consistent with (4), until a decision threshold was crossed (Tuerlinckx et al., 2001). Accumulation continued until all evidence measurements had been processed, at which point a simulated confidence was determined in accordance with (54). Full details of the simulation can be found in Supplement Section G.

Fig. 8 shows accuracy against confidence for two cases, one in which there is no variability in drift-rate scaling, $\varphi$, and one with variability. Full details of the process used to plot the data are also provided in Supplement Section G. Only one approximation is relevant when there is no variability in drift-rate scaling, approximation (a). This approximation appears to have little effect on the mapping

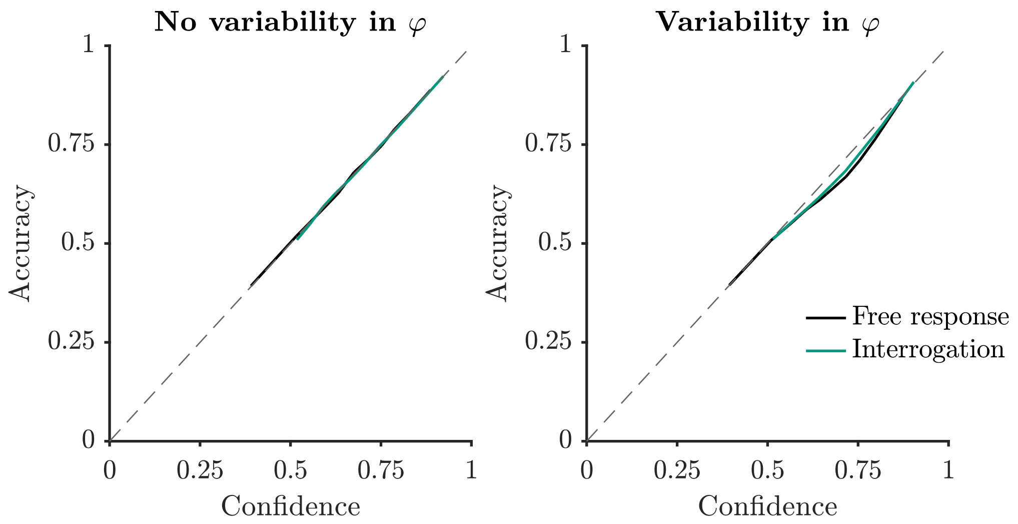


Figure 8: Accuracy as a function of confidence, where confidence is calculated using the observer's generative model of their evidence measurements. This model only approximates the true generative model. When there is variability in drift-rate scaling, the approximations cause the observer's confidence to show some deviations from calibration. Simulation and plotting details in Supplement Section G.

between accuracy and confidence. An additional approximation is relevant when there is variability in drift-rate scaling, assumption (b). We can see that this causes a moderate mismatch between accuracy and confidence (Fig. 8).

It is interesting to note that the latter approximation, that observers ignore the effect of drift rate on stimulus variability, is implicitly present in much previous work. This is because stochastically fluctuating stimuli are often used, but stimulus variability is not treated separately from accumulator variability in the computational models applied (e.g. Kiani et al., 2014; Ratcliff and McKoon, 2008; van den Berg et al., 2016, although see Zylberberg et al., 2016). Fig. 8 suggests this approximation does not cause large issues.

# C Bayesian observer's policy

At some time $t$, the observer has gathered $N$ sensory samples, $\delta x_1, \delta x_2, ..., \delta x_N$, collectively denoted $\boldsymbol{\delta x}$. The samples are generated according to (6), (7), and (8). Using Bayes rule the observer can infer the probability of the two possible values for $S$. Using a result from Drugowitsch et al. (2012), Moran (2015) derived the posterior probability over the two options $S = 1$ and $S = 2$, for exactly the case we have. For completeness, we rederive the posterior here.

Given all the evidence measurements so far,

$$p(S|\boldsymbol{\delta x}) \propto p(\boldsymbol{\delta x}|S)p(S) \tag{55}$$

$$\propto \int \prod_{i=1}^{N} p(\delta x_i|\nu)p(\nu|S)d\nu\,, \tag{56}$$

where we have used the flat prior over $S$ in (1). Looking at the product in (56), using (8), and using standard formulae for the product of normal distributions (e.g. see Bromiley, 2014) we have,

$$\prod_{i=1}^{N} p(\delta x_i|\nu) = \prod_{i=1}^{N} N(\delta x_i; \nu\delta t, s^2\delta t) \tag{57}$$

$$\propto N(\nu; \frac{x}{t}, \frac{s^2}{t})\,, \tag{58}$$

where the proportionality sign indicates proportionality with respect to $\nu$, $x = \sum_{i=i}^{N} \delta x_i$, and we have used that $t = \sum_{i=1}^{N} \delta t$. Hence in (56) we have,

$$p(S|\boldsymbol{\delta x}) \propto \int N(\nu; \frac{x}{t}, \frac{s^2}{t})N(\nu; \pm\nu_0, \sigma_\nu^2)d\nu\,, \tag{59}$$

where the sign on $\nu_0$ is determined by $S$. Using the result in Supplement Section A,

$$p(S|\boldsymbol{\delta x}) \propto N(\pm\nu_0; \frac{x}{t}, \frac{s^2}{t} + \sigma_\nu^2)\,. \tag{60}$$

Rearranging, we find the (not scaled) log-posterior ratio is given by,

$$\log \frac{p(S=2|\boldsymbol{\delta x})}{p(S=1|\boldsymbol{\delta x})} = \frac{2x\nu_0}{s^2 + t\sigma_\nu^2}\,. \tag{61}$$

# D Effect of posterior ratio scaling

In the main text we considered a model in which the observer uses a noisy readout of the scaled log-posterior ratio to determine their confidence. A noisy readout of the scaled log-posterior ratio ($x_{lp}$), is equivalent to a scaled version of a noisy readout of the unscaled log-posterior ratio ($x_{lp}/K$). First consider the readout in the main text in (10):

$$p(x_c|x_{lp}) = N(x_c; x_{lp}, \sigma_m^2)\,. \tag{62}$$

Then consider a noisy readout of the same form but of the unscaled log-posterior ratio. Denote this new readout $\widetilde{x}_c$. We have,

$$p(\widetilde{x}_c|\frac{x_{lp}}{K}) = N(\widetilde{x}_c; \frac{x_{lp}}{K}, \widetilde{\sigma}_m^2)\,, \tag{63}$$

where $\widetilde{\sigma}_m$ is the standard deviation of the metacognitive noise affecting this readout.

Our claim is that the distribution over $\widetilde{x}_c$ when scaled by K, is identical to the distribution over $x_c$. Consider a scaled version of $\widetilde{x}_c$, $\widehat{x}_c = K\widetilde{x}_c$. Using the rule for changing the variable of a probability density function, where the relationship between the variables being swapped is monotonic, then,

$$\begin{aligned}
p(\widehat{x}_c|\frac{x_{lp}}{K}) &= \left|\frac{\mathrm{d}\widetilde{x}_c}{\mathrm{d}\widehat{x}_c}\right| N(\widetilde{x}_c; \frac{x_{lp}}{K}, \widetilde{\sigma}_m^2) && (64)\\
&= \frac{1}{K}\frac{1}{\sqrt{2\pi\widetilde{\sigma}_m^2}}\exp\left\{-\frac{(\widetilde{x}_c - \frac{x_{lp}}{K})^2}{2\widetilde{\sigma}_m^2}\right\} && (65)\\
&= \frac{1}{\sqrt{2\pi K^2\widetilde{\sigma}_m^2}}\exp\left\{-\frac{(K\widetilde{x}_c - x_{lp})^2}{2K^2\widetilde{\sigma}_m^2}\right\} && (66)\\
&= N(\widehat{x}_c; x_{lp}, K^2\widetilde{\sigma}_m^2)\,. && (67)
\end{aligned}$$

Hence, the scaled readout of the unscaled log-posterior ratio, $\widehat{x}_c$, has the same distribution as the readout of the scaled log-posterior ratio, $x_c$. The metacognitive noise parameters in the two cases are related to each other through $\sigma_m = K\widetilde{\sigma}_m$.

## E Details of interrogation condition derivation

The steps leading to (11) were described in words in the main text. In equations, these steps are as follows.

$$p(C=i|R=2,\boldsymbol{E}) = \int_{d_i}^{d_{i+1}} dx_c \int dx_{lp}\ p(x_c|x_{lp})p(x_{lp}|R=2,\boldsymbol{E}) \tag{68}$$

$$= \int_{d_i}^{d_{i+1}} dx_c \int dx_{lp}\ \frac{p(x_c|x_{lp})p(R=2|x_{lp})p(x_{lp}|\boldsymbol{E})}{p(R=2|\boldsymbol{E})}\,. \tag{69}$$

For now just consider $p(x_{lp}|\boldsymbol{E})$, which we need to find (12). Let's marginalise over $\varphi$,

$$p(x_{lp}|\boldsymbol{E}) = \int d\varphi\ p(x_{lp}|\varphi,\boldsymbol{E})p(\varphi|\boldsymbol{E})\,. \tag{70}$$

$\boldsymbol{E}$ and $\varphi$ are independent of each other (when not conditioned on other variables; see Fig. 4). Hence,

$$p(\varphi|\boldsymbol{E}) = p(\varphi) = N(\varphi;1,\sigma_\varphi^2)\,, \tag{71}$$

where we have used (5).

Recall from (4) that,

$$p(\delta x_i|\delta E_i,\varphi) = N(\delta x_i;\delta E_i\varphi,\sigma_{acc}^2\delta t)\,. \tag{72}$$

Using standard formulae for the sum of normally distributed random variables, we have that $x=\sum_i \delta x_i$ will be given by,

$$p(x|\varphi,\boldsymbol{E}) = N(x;E\varphi,t_e\sigma_{acc}^2)\,. \tag{73}$$

Using (9) we have,

$$p(x_{lp}|\varphi,\boldsymbol{E}) = N(x_{lp};\frac{E\varphi}{\theta(t_e)},\frac{t_e\sigma_{acc}^2}{\theta^2(t_e)})\,. \tag{74}$$

Substituting these results into (70) gives us,

$$p(x_{lp}|\boldsymbol{E}) = \int d\varphi\ N(x_{lp};\frac{E\varphi}{\theta(t_e)},\frac{t_e\sigma_{acc}^2}{\theta^2(t_e)})N(\varphi;1,\sigma_\varphi^2) \tag{75}$$

$$= \frac{\theta(t_e)}{E}\int d\varphi\ N(\varphi;\frac{x_{lp}\theta(t_e)}{E},\frac{t_e\sigma_{acc}^2}{E^2})N(\varphi;1,\sigma_\varphi^2)\,. \tag{76}$$

We can apply the result in Supplement Section A. Simplifying, we find,

$$p(x_{lp}|\boldsymbol{E}) = N(x_{lp};\frac{E}{\theta(t_e)},\frac{E^2\sigma_\varphi^2+t_e\sigma_{acc}^2}{\theta^2(t_e)})\,. \tag{77}$$

Returning to (12), we now have,

$$\Psi(x_c) = \int dx_{lp}\ p(x_c|x_{lp})p(R|x_{lp})N(x_{lp};\frac{E}{\theta(t_e)},\frac{E^2\sigma_\varphi^2+t_e\sigma_{acc}^2}{\theta^2(t_e)})\,. \tag{78}$$

In the case in which we have no metacognitive noise, $x_c = x_{lp}$. We can express this using the Dirac delta function as $p(x_c|x_{lp}) = \delta(x_c - x_{lp})$. Then we have,

$$\Psi(x_c) = \int dx_{lp}\ \delta(x_c-x_{lp})p(R|x_{lp})N(x_{lp};\frac{E}{\theta(t_e)},\frac{E^2\sigma_\varphi^2+t_e\sigma_{acc}^2}{\theta^2(t_e)}) \tag{79}$$

$$= p(R|x_{lp}=x_c)N(x_c;\frac{E}{\theta(t_e)},\frac{E^2\sigma_\varphi^2+t_e\sigma_{acc}^2}{\theta^2(t_e)})\,, \tag{80}$$

Using (11) and our expression for $\Psi(x_c)$,

$$p(C=i|R=2,\boldsymbol{E}) = \frac{1}{p(R=2|\mathbf{E})}\int_{d_i}^{d_{i+1}} dx_c\ p(R=2|x_{lp}=x_c)N(x_c;\frac{E}{\theta(t_e)},\frac{E^2\sigma_\varphi^2+t_e\sigma_{acc}^2}{\theta^2(t_e)}) \quad (81)$$

$$= \frac{1}{p(R=2|\mathbf{E})}\int_{\max\{0,d_i\}}^{\max\{0,d_{i+1}\}} dx_c\ N(x_c;\frac{E}{\theta(t_e)},\frac{E^2\sigma_\varphi^2+t_e\sigma_{acc}^2}{\theta^2(t_e)})\,, \quad (82)$$

where we have used that $p(R=2|x_{lp}=x_c)$ is zero anywhere $x_{lp}$ is inconsistent with the response $R=2$ (i.e. $x_{lp}<0$), and one elsewhere. In the case where $R=1$ an identical expression holds, except the limits become $\min\{0,-d_{i+1}\}$ and $\min\{0,-d_i\}$, and we divide by $p(R=1|\mathbf{E})$ not $p(R=2|\mathbf{E})$.

We can compute the probability of the response, $p(R|\mathbf{E})$, using,

$$p(R|\boldsymbol{E}) = \int_{-\infty}^{\infty} dx_{lp}\ p(R|x_{lp})p(x_{lp}|\boldsymbol{E}) \quad (83)$$

$$= \int_a^b dx_{lp}\ N(x_{lp};\frac{E}{\theta(t_e)},\frac{E^2\sigma_\varphi^2+t_e\sigma_{acc}^2}{\theta^2(t_e)}) \quad (84)$$

Where $a=-\infty, b=0$ for $R=1$ or $a=0, b=\infty$ for $R=2$. These limits again come from using that $p(R|x_{lp})=1$ when $R$ and $x_{lp}$ are consistent, and $p(R|x_{lp})=0$ otherwise.

Let's now consider what happens in the presence of metacognitive noise, when $x_c$ no longer equals $x_{lp}$, but is a noisy version of it as in (10). Returning to (78), in this case $\Psi(x_c)$ becomes,

$$\Psi(x_c) = \int dx_{lp}\ N(x_c;x_{lp},\sigma_m^2)p(R|x_{lp})N(x_{lp};\frac{E}{\theta(t_e)},\frac{E^2\sigma_\varphi^2+t_e\sigma_{acc}^2}{\theta^2(t_e)})\,. \quad (85)$$

As discussed in the main text, the product of $p(R|x_{lp})$ and the normal distribution over $x_{lp}$ is a normal distribution truncated to the region where $R$ and $x_{lp}$ are consistent, because $p(R|x_{lp})$ is one when $R$ is consistent with $x_{lp}$, and zero otherwise. Note that the product of these two terms is not a probability distribution over $x_{lp}$ because it is not normalised. However, we can write this product as a scaled truncated normal distribution. Using $J(x_{lp})$ as an abbreviation,

$$J(x_{lp}) = p(R|x_{lp})N(x_{lp};\frac{E}{\theta(t_e)},\frac{E^2\sigma_\varphi^2+t_e\sigma_{acc}^2}{\theta^2(t_e)}) \quad (86)$$

$$= TN(x_{lp};\mu_{lp},\sigma_{lp}^2,a,b)\int_a^b dx'_{lp}\ N(x'_{lp};\frac{E}{\theta(t_e)},\frac{E^2\sigma_\varphi^2+t_e\sigma_{acc}^2}{\theta^2(t_e)}) \quad (87)$$

$$= TN(x_{lp};\mu_{lp},\sigma_{lp}^2,a,b)p(R|\boldsymbol{E})\,. \quad (88)$$

We have used (84) to get the final line. As before $a=-\infty, b=0$ or $a=0, b=\infty$ depending on the response made. $\mu_{lp}$ and $\sigma_{lp}^2$, are the mean and variance of the distribution prior to truncation (see equations 15 and 16). (Parameterisation of the truncated normal distribution, $TN$, is described in the main text.)

Returning to (85) we now have,

$$\Psi(x_c) = p(R|\boldsymbol{E})\int_{-\infty}^{\infty} dx_{lp}\ N(x_c;x_{lp},\sigma_m^2)TN(x_{lp};\mu_{lp},\sigma_{lp}^2,a,b)\,. \quad (89)$$

Consider a change of variables $y=x_c-x_{lp}$,

$$\Psi(x_c) = -p(R|\boldsymbol{E})\int_{\infty}^{-\infty} dy\ N(y;0,\sigma_m^2)TN(x_c-y;\mu_{lp},\sigma_{lp}^2,a,b) \quad (90)$$

$$= p(R|\boldsymbol{E})\int_{-\infty}^{\infty} dy\ N(y;0,\sigma_m^2)TN(x_c-y;\mu_{lp},\sigma_{lp}^2,a,b)\,. \quad (91)$$

This expression is the convolution of a normal distribution and a truncated normal distribution. We can perform the convolution by writing the distributions out in full, collecting all terms containing $y$ into a

single exponential, and integrating (S. Turban, personal communication, December, 2019):

$$\begin{aligned}\Psi(x_c) &= p(R|\boldsymbol{E})\int_{-\infty}^{\infty} dy\ N(y;0,\sigma_m^2)TN(x_c-y;\mu_{lp},\sigma_{lp}^2,a,b) &(92)\\ &= p(R|\boldsymbol{E})\int_{-\infty}^{\infty} dy\frac{1}{2\pi\sqrt{\sigma_m^2\sigma_{lp}^2}}\frac{1}{\Phi(\frac{b-\mu_{lp}}{\sigma_{lp}})-\Phi(\frac{a-\mu_{lp}}{\sigma_{lp}})}e^{-\frac{y^2}{2\sigma_m^2}}e^{-\frac{(x_c-y-\mu_{lp})^2}{2\sigma_{lp}^2}}\mathbb{1}_{y\in[x_c-b,x_c-a]} &(93)\\ &= p(R|\boldsymbol{E})\frac{1}{\Phi(\frac{b-\mu_{lp}}{\sigma_{lp}})-\Phi(\frac{a-\mu_{lp}}{\sigma_{lp}})}N(x_c;\mu_{lp},\sigma_m^2+\sigma_{lp}^2)\Big[\Phi(\frac{x_c-a-\alpha}{\beta})-\Phi(\frac{x_c-b-\alpha}{\beta})\Big] &(94)\end{aligned}$$

where $\Phi()$ indicates the standard cumulative normal distribution, $\mathbb{1}$ is an indicator function (which is one when its argument is true, and zero otherwise), $\alpha=\frac{\beta^2(x_c-\mu_{lp})}{\sigma_{lp}^2}$ and $\beta^2=\frac{\sigma_m^2\sigma_{lp}^2}{\sigma_m^2+\sigma_{lp}^2}$.

In our case, $a$ and $b$ take very specific values. Let,

$$L=\begin{cases}-1 & \text{if } a=-\infty, b=0;\ \text{i.e. R=1}\\ 1 & \text{if } a=0, b=\infty;\ \text{i.e. R=2}\,,\end{cases}$$

then we can write,

$$\Psi(x_c)=p(R|\boldsymbol{E})\frac{1}{\Phi(L\frac{\mu_{lp}}{\sigma_{lp}})}N(x_c;\mu_{lp},\sigma_m^2+\sigma_{lp}^2)\Phi\left(L\frac{x_c\sigma_{lp}^2+\mu_{lp}\sigma_m^2}{\sigma_m\sigma_{lp}\sqrt{\sigma_m^2+\sigma_{lp}^2}}\right), \tag{95}$$

and using (11) our result for confidence is,

$$p(C=i|R,\boldsymbol{E})=L\int_{Ld_i}^{Ld_{i+1}} dx_c\ \frac{1}{\Phi(L\frac{\mu_{lp}}{\sigma_{lp}})}N(x_c;\mu_{lp},\sigma_m^2+\sigma_{lp}^2)\Phi\left(L\frac{x_c\sigma_{lp}^2+\mu_{lp}\sigma_m^2}{\sigma_m\sigma_{lp}\sqrt{\sigma_m^2+\sigma_{lp}^2}}\right). \tag{96}$$

We have also used $L$ in the above expression to account for the different limits of integration when $R=1$ and $R=2$.

We can solve the integral in (96) via numerical integration. It is also possible to rearrange the expression to a form that is faster to evaluate numerically. Consider a change of variables,

$$z=\frac{x_c-\mu_{lp}}{\sqrt{\sigma_m^2+\sigma_{lp}^2}}\,. \tag{97}$$

Then we have,

$$dz=\frac{dx_c}{\sqrt{\sigma_m^2+\sigma_{lp}^2}} \tag{98}$$

and,

$$\begin{aligned}N(x_c;\mu_{lp},\sigma_m^2+\sigma_{lp}^2) &= \frac{1}{\sqrt{\sigma_m^2+\sigma_{lp}^2}}N(z;0,1) &(99)\\ &= \frac{1}{\sqrt{\sigma_m^2+\sigma_{lp}^2}}\phi(z)\,, &(100)\end{aligned}$$

where $\phi()$ indicates the standard normal distribution. Also denote,

$$\begin{aligned}g &= \frac{L\mu_{lp}\sqrt{\sigma_m^2+\sigma_{lp}^2}}{\sigma_m\sigma_{lp}}=L\frac{\mu_{lp}\sigma_{lp}^2+\mu_{lp}\sigma_m^2}{\sigma_m\sigma_{lp}\sqrt{\sigma_m^2+\sigma_{lp}^2}} &(101)\\ h &= \frac{L\sigma_{lp}}{\sigma_m}=\frac{L\sigma_{lp}^2}{\sigma_m\sigma_{lp}}\,. &(102)\end{aligned}$$

Then,

$$g + hz = L\frac{x_c\sigma_{lp}^2 + \mu_{lp}\sigma_m^2}{\sigma_m\sigma_{lp}\sqrt{\sigma_m^2 + \sigma_{lp}^2}} \,. \tag{103}$$

Hence, in (96) we have,

$$p(C = i|R, \boldsymbol{E}) = L\frac{1}{\Phi(L\frac{\mu_{lp}}{\sigma_{lp}})}\int_{e_i}^{e_{i+1}} dz\ \phi(z)\Phi(g + hz)\,, \tag{104}$$

where,

$$e_i = \frac{Ld_i - \mu_{lp}}{\sqrt{\sigma_m^2 + \sigma_{lp}^2}} \,. \tag{105}$$

Using result 10010.1 from Owen (1980), gives us,

$$p(C = i|R, \boldsymbol{E}) = L\frac{1}{\Phi(L\frac{\mu_{lp}}{\sigma_{lp}})}\Bigg[ \quad BvN\Big(\frac{g}{\sqrt{1+h^2}}, e_{i+1}; \frac{-h}{\sqrt{1+h^2}}\Big) \tag{106}$$

$$- \quad BvN\Big(\frac{g}{\sqrt{1+h^2}}, e_i; \frac{-h}{\sqrt{1+h^2}}\Big)\Bigg]\,, \tag{107}$$

where,

$$BvN(l_1, l_2, \rho) = \int_{-\infty}^{l_1}\int_{-\infty}^{l_2} \frac{1}{2\pi\sqrt{1-\rho^2}} e^{-\frac{1}{2(1-\rho^2)}(x^2+y^2-2xy\rho)} dxdy\,, \tag{108}$$

is the bivariate cumulative normal distribution, corresponding to a distribution with mean and covariance,

$$\mu = \begin{bmatrix} 0 \\ 0 \end{bmatrix} \tag{109}$$

$$\Sigma = \begin{bmatrix} 1 & \rho \\ \rho & 1 \end{bmatrix} \,. \tag{110}$$

This integral can then be numerically evaluated using standard functions.

# F Details of free response condition derivation

In this section we cover certain details of the derivations for the free response condition, starting with how we arrived at the distribution over $\varphi$ in (26).

For the purpose of inferring $\varphi$, we approximate the evidence stream, $\boldsymbol{E}$, by its average prior to the time of the decision, $\overline{E}$,

$$\begin{aligned} p(\varphi|R, t_r, \boldsymbol{E}) &= p(\varphi|x_d, t_d, \boldsymbol{E}) && (111) \\ &= p(\varphi|x_d, t_d, \overline{E}), && (112) \end{aligned}$$

where we have used (25) in the first line. Precisely, $\overline{E}$ is given by $\overline{E} = \sum_i \delta E_i / t_d$, where the summation is taken over all time steps prior to the decision.

Using Bayes rule,

$$\begin{aligned} p(\varphi|R, t_r, \boldsymbol{E}) &= p(\varphi|x_d, t_d, \overline{E}) && (113) \\ &\propto p(x_d, t_d|\varphi, \overline{E}) p(\varphi|\overline{E}) && (114) \\ &\propto p(\varphi|\overline{E}) \sum_{\omega \in \Omega} p(\boldsymbol{\delta x}^{(\omega)}|\varphi, \overline{E}) && (115) \\ &\propto p(\varphi|\overline{E}) \sum_{\omega \in \Omega} \prod_i p(\delta x_i^{(\omega)}|\varphi, \overline{E}). && (116) \end{aligned}$$

In this equation, $\Omega$ is the set of all paths that do not cross the decision threshold prior to $t_d$, and arrive at the threshold at $t_d$ (Moreno-Bote, 2010). $\boldsymbol{\delta x}^{(\omega)}$ is the $\boldsymbol{\delta x}$ vector for the particular path $\omega$.

As discussed in the main text, we also approximate $p(\varphi|\overline{E}) \approx p(\varphi)$. The approximation gives,

$$p(\varphi|R, t_r, \boldsymbol{E}) \propto p(\varphi) \sum_{\omega \in \Omega} \prod_i p(\delta x_i^{(\omega)}|\varphi, \overline{E}). \quad (117)$$

Returning to (4) but now using our approximation replacing the full evidence stream with the average evidence, we have that $\delta x_i$ is given by

$$\begin{aligned} p(\delta x_i|\delta E_i, \varphi) &\approx p(\delta x_i|\overline{E}, \varphi) && (118) \\ &\approx N(\delta x_i; \varphi \overline{E} \delta t, \sigma_{acc}^2 \delta t) && (119) \\ &\propto N(\varphi; \frac{\delta x_i}{\overline{E} \delta t}, \frac{\sigma_{acc}^2}{\overline{E}^2 \delta t}). && (120) \end{aligned}$$

The proportionality is with respect to $\varphi$.

Using standard results for the product of normal distributions we find

$$\begin{aligned} \prod_i p(\delta x_i|\overline{E}, \varphi) &\propto \prod_i N(\varphi; \frac{\delta x_i}{\overline{E} \delta t}, \frac{\sigma_{acc}^2}{\overline{E}^2 \delta t}) && (121) \\ &\propto N(\varphi; \frac{x_d}{\overline{E} t_d}, \frac{\sigma_{acc}^2}{\overline{E}^2 t_d}) && (122) \\ &= K^{(\omega)} N(\varphi; \frac{x_d}{\overline{E} t_d}, \frac{\sigma_{acc}^2}{\overline{E}^2 t_d}), && (123) \end{aligned}$$

where we have used $t_d = \sum \delta t$, $x_d = \sum \delta x_i$, and $K^{(\omega)}$ is the constant of proportionality (see Drugowitsch et al., 2012 for closely related calculations). This constant will be different for different paths, so we explicitly indicate the path, $\omega$. Hence, we have in (117)

$$\begin{aligned} p(\varphi|R, t_r, \boldsymbol{E}) &\propto p(\varphi) \sum_{\omega \in \Omega} K^{(\omega)} N(\varphi; \frac{x_d}{\overline{E} t_d}, \frac{\sigma_{acc}^2}{\overline{E}^2 t_d}) && (124) \\ &\propto p(\varphi) N(\varphi; \frac{x_d}{\overline{E} t_d}, \frac{\sigma_{acc}^2}{\overline{E}^2 t_d}). && (125) \end{aligned}$$

This is the expression given in the main text that we were aiming to derive. However, we can simplify this expression further.

Recalling (5), $\varphi$ is distributed as,

$$p(\varphi) = N(\varphi; 1, \sigma_\varphi^2)\,, \tag{126}$$

giving

$$\begin{aligned} p(\varphi|R, t_r, \boldsymbol{E}) &\propto N(\varphi; 1, \sigma_\varphi^2) N(\varphi; \frac{x_d}{\overline{E} t_d}, \frac{\sigma_{acc}^2}{\overline{E}^2 t_d}) & (127) \\ &= N\Big(\varphi; \frac{\sigma_{acc}^2 + x_d \sigma_\varphi^2 \overline{E}}{\sigma_{acc}^2 + t_d \sigma_\varphi^2 \overline{E}^2}, \frac{\sigma_{acc}^2 \sigma_\varphi^2}{\sigma_{acc}^2 + t_d \sigma_\varphi^2 \overline{E}^2}\Big)\,, & (128) \end{aligned}$$

where we have used standard formulae for the product of normal distributions.

In the main text we derived an expression for the first term in the integral in (23), and in this supplement section we have derived an expression for the second term. These expressions can be used to perform the integral in (23) as follows,

$$\begin{aligned} p(x_{lp}|R, t_r, \boldsymbol{E}) &= \int d\varphi\; p(x_{lp}|\varphi, R, t_r, \boldsymbol{E}) p(\varphi|R, t_r, \boldsymbol{E}) & (129) \\ &= \int d\varphi\; N(x_{lp}; \frac{x_d + \varphi \Delta E}{\theta(t_e)}, \frac{\sigma_{acc}^2 I}{\theta^2(t_e)}) N\Big(\varphi; \frac{\sigma_{acc}^2 + x_d \sigma_\varphi^2 \overline{E}}{\sigma_{acc}^2 + t_d \sigma_\varphi^2 \overline{E}^2}, \frac{\sigma_{acc}^2 \sigma_\varphi^2}{\sigma_{acc}^2 + t_d \sigma_\varphi^2 \overline{E}^2}\Big) & (130) \\ &= \frac{\theta(t_e)}{\Delta E} \int d\varphi\; N(\varphi; \frac{\theta(t_e) x_{lp} - x_d}{\Delta E}, \frac{\sigma_{acc}^2 I}{\Delta E^2}) N\Big(\varphi; \frac{\sigma_{acc}^2 + x_d \sigma_\varphi^2 \overline{E}}{\sigma_{acc}^2 + t_d \sigma_\varphi^2 \overline{E}^2}, \frac{\sigma_{acc}^2 \sigma_\varphi^2}{\sigma_{acc}^2 + t_d \sigma_\varphi^2 \overline{E}^2}\Big) & (131) \end{aligned}$$

Applying the result in Supplement Section A and rearranging we produce (30).

# G Simulation and plotting details

We simulated the task described in the main text with the parameters shown in Table 4. The only difference between the simulation and the setup described in the main text is that evidence in a frame could not go below 0 or above 3096. Evidence within a frame was resampled until it met these constraints. The midpoint between the means of the two evidence signals, referred to as "reference value" and denoted $\overline{\mu}/2$, was sampled each trial. It was sampled from a normal distribution centred on 1000, with standard deviation of 100, that was truncated at 500 and 1500.

| Variable | Expression | Simulation value |
|---|---|---|
| Duration of a frame | $t_f$ | 50ms |
| Max evidence in frame | | 3096 |
| Min evidence in frame | | 0 |
| Reference value | $p(\overline{\mu}/2)$ | See text |
| Mean difference between evidence signals | $\Delta\mu$ | 90 |
| Standard deviation of evidence in a frame | $\sigma_E$ | 311 |

Table 4: Parameters for the task used in the simulation.

The parameters used for the observer are shown in Table 5. Within a simulation, all participants were simulated using the same parameters. Except for Fig. 7, half of the trials were simulated for the free response condition, and half for the interrogation condition. For Fig. 7 all trials were from the free response condition, but two different versions of the free response condition were used, as described in the main text.

A linear decision threshold, symmetric for both options, was used in the free response condition. At the onset of evidence accumulation the threshold was at the value given in Table 5 for threshold height, and it subsequently changed at the rate given by threshold slope. In the interrogation condition, stimulus presentation duration was drawn from a normal distribution of mean 0.8 s and standard deviation 0.3 s, truncated at 0.2 and 4 s.

| Variable | Expression | Fig. 8 | Fig. 6 | Fig. 7 |
|---|---|---|---|---|
| Participants per simulation | | 40 | 40 | 40 |
| Trials per participant | | 12800 | 12800 | 64000 |
| Pipeline duration | $I$ | 350 ms | 350 ms | 100ms |
| Standard deviation of accumulator noise | $\sigma_{acc}$ | 2860 | 2860 | 2860 |
| Standard deviation of drift-rate scaling | $\sigma_{\varphi}$ | 1 or 0 (separate simulations) | 1 or 0 (separate simulations) | 0.6 |
| Standard deviation of metacognitive noise | $\sigma_m$ | 0 | 1500 | 1500 |
| Threshold height | | 2000 | 2000 | 2000 |
| Threshold slope | | 0 | $-800\ s^{-1}$ | 0 |

Table 5: Parameters of the simulated observers.

The evolution of the observer's accumulator was simulated using small time steps of duration 0.1 ms. Each accumulator increment was determined by sampling from the distribution in (4). Simulation of the accumulation continued until a decision threshold was crossed, and all remaining evidence in the pipeline had been processed. The only exception was the simulation for Fig. 7. For the "Immediate

conf." condition in this plot, accumulation terminated as soon as the decision threshold was reached, consistent with the idea that the manipulation used by Kiani et al. (2014) ensured confidence was not based on pipeline evidence.

Confidence reports were produced in accordance with the model (see (54), (10) and Fig. 2). Where a plot refers to binned confidence, the confidence values produced by the model have been divided into 10 bins of equal numbers of data points on a participant-by-participant basis.

To make predictions for confidence using the derived expressions, we set the values of the parameters in these expressions ($\sigma_{acc}$, $\sigma_{\varphi}$, $\sigma_m$, $I$, threshold height and threshold slope) to the true values used to simulate the data. We set the bin edge parameters ($d_i$) to the values that were used for dividing the simulated confidence reports into 10 bins.

Prior to plotting, data for the x-axis was binned separately for each participant and data series. For each participant, data series, and bin, the average value of the x-variable was computed. Using these averages, the average value across participants was computed, and this determined the x-location of each bin. 10 bins were used apart from in Fig. 7 where 5 bins were used. The y-variable was calculated separately for each participant and bin, before averaging across participants. Error bars, and the width of error shading, represent $\pm 1$ standard error of the mean across participants.

# H Testing the approximations in other models

In Fig. 6 we compared the mean and variance of confidence produced via simulations, and confidence produced through the predictions derived in the main text. The aim was to test whether the approximations made during the derivations were reasonable. We found little discrepancy between the simulated and predicted confidence. For completeness, we ran a similar analysis using a model not considered in the main text. Specifically, we considered an observer who did not use a Bayesian readout for confidence. Instead their confidence was directly determined by the final state of the accumulator: More accumulated evidence favouring the choice generated higher confidence. We adjusted the reported derivations to cover this case by setting $\theta(t) = 1$ (see equation 9).

For this model we found similar discrepancies between the simulations and the derived predictions. Figure 9 provides an example. Again, discrepancies were in general small. Nevertheless, we should bear in mind that just because an approximation works well for some model, or at some parameter values, does not mean it is guaranteed to work well in all models or at all parameter values.

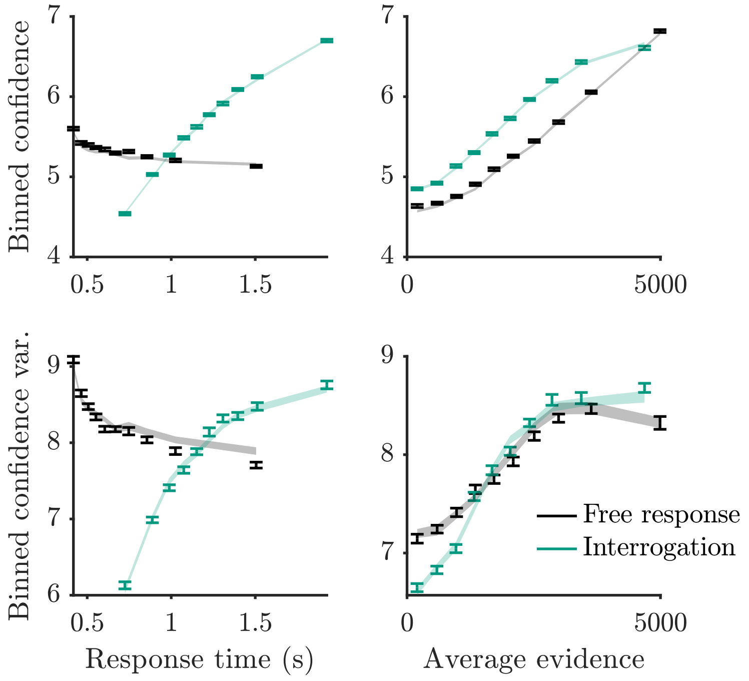


Figure 9: Mean and variance of binned confidence, produced via simulation of an alternative model (error bars), and produced through the derived predictions adjusted for an alternative model (shading). The alternative model featured confidence that was a direct readout of the evidence accumulated, rather than a Bayesian readout for confidence. "Average evidence" here refers to the absolute value of the following quantity: The difference in dots summed over all presented frames, divided by the duration of stimulus presentation.

# References


Bromiley, P. A. (2014). *Products and convolutions of gaussian probability density functions*. Retrieved 2020, from http://www.tina-vision.net/docs/memos/2003-003.pdf

Drugowitsch, J., Moreno-Bote, R., Churchland, A. K., Shadlen, M. N., & Pouget, A. (2012). The cost of accumulating evidence in perceptual decision making. *Journal of Neuroscience*, *32*(11), 3612–3628. https://doi.org/10.1523/JNEUROSCI.4010-11.2012

Kiani, R., Corthell, L., & Shadlen, M. N. (2014). Choice certainty is informed by both evidence and decision time. *Neuron*, *84*(6), 1329–1342. https://doi.org/10.1016/j.neuron.2014.12.015

Kiani, R., & Shadlen, M. N. (2009). Representation of confidence associated with a decision by neurons in the parietal cortex. *Science*, *324*(5928), 759–764. https://doi.org/10.1126/science.1169405

Moran, R. (2015). Optimal decision making in heterogeneous and biased environments. *Psychonomic Bulletin & Review*, *22*(1), 38–53. https://doi.org/10.3758/s13423-014-0669-3

Moreno-Bote, R. (2010). Decision confidence and uncertainty in diffusion models with partially correlated neuronal integrators. *Neural computation*, *22*(7), 1786–1811. https://doi.org/10.1162/neco.2010.12-08-930

Owen, D. B. (1980). A table of normal integrals: A table. *Communications in Statistics - Simulation and Computation*, *9*(4), 389–419. https://doi.org/10.1080/03610918008812164

Ratcliff, R., & McKoon, G. (2008). The diffusion decision model: Theory and data for two-choice decision tasks. *Neural Computation*, *20*(4), 873–922. https://doi.org/10.1162/neco.2008.12-06-420

Tuerlinckx, F., Maris, E., Ratcliff, R., & De Boeck, P. (2001). A comparison of four methods for simulating the diffusion process. *Behavior Research Methods, Instruments, & Computers*, *33*(4), 443–456. https://doi.org/10.3758/BF03195402

van den Berg, R., Anandalingam, K., Zylberberg, A., Kiani, R., Shadlen, M. N., & Wolpert, D. M. (2016). A common mechanism underlies changes of mind about decisions and confidence. *eLife*, *5*, 1–21. https://doi.org/10.7554/eLife.12192

Zylberberg, A., Fetsch, C. R., & Shadlen, M. N. (2016). The influence of evidence volatility on choice, reaction time and confidence in a perceptual decision. *eLife*, *5*. https://doi.org/10.7554/eLife.17688
